# A Pathology‐Instructed Theranostic Platform with Mechanoadaptive and ROS‐Powered Nanobreathing Functions for Precision Myocardial Repair

**DOI:** 10.1002/advs.76312

**Published:** 2026-07-02

**Authors:** Zheng Luo, Cui Yang, Liuzhou Mao, Panqin Ma, Chiyu Jia, Karen Yuanting Tang, Xian Jun Loh, Yun‐Long Wu

**Affiliations:** ^1^ Center of Burn & Plastic and Wound Healing Surgery, The First Affiliated Hospital of University of South China Hengyang Medical School, University of South China Hengyang Hunan China; ^2^ State Key Laboratory of Vaccines for Infectious Diseases Xiang An Biomedicine Laboratory, Fujian Provincial Key Laboratory of Innovative Drug Target Research School of Pharmaceutical Sciences, Faculty of Medicine and Life Sciences Xiamen University Xiamen China; ^3^ Institute of Materials Research and Engineering (IMRE) Agency for Science, Technology and Research (A*STAR) Singapore Republic of Singapore; ^4^ School of Medicine Xiamen University Xiamen China

**Keywords:** Enzyme therapy, Mechanoadaptive protein hydrogel, Mitochondrial antioxidation, Multimodal imaging, Myocardial infarction

## Abstract

Current myocardial infarction (MI) biomaterials often fail to dynamically adapt to the evolving pathological microenvironment (e.g., ROS bursts, hypoxia), lacking both mechanical compatibility and in situ feedback. To overcome these limitations, we developed an albumin hydrogel platform (BST) with a closed‐loop pathological response system. BST is a pH‐responsive hydrogel, functionalized with MRI/CT probes and loaded with mitochondria‐targeted CAT‐SOD enzyme nanogels (CSDT). It forms a self‐repairing scaffold with high shear viscosity (∼300 Pa·s), elasticity, and cardiac‐like mechanics (∼7.5 kPa), stabilizing the infarct wall. Hypoxia‐induced acidosis triggers the release of CSDT nanogels and albumin, enabling mitochondrial ROS scavenging and O_2_ generation, while albumin restores tissue osmotic balance. These actions collectively alleviate oxidative stress, modulate immune responses, and promote cardiomyocyte survival by enhancing autophagy and anti‐apoptotic pathways. This inside‐out feedback mechanism reverses oxidative stress and hypoxia, shifts macrophages to a reparative M2 phenotype, and increases angiogenesis by ∼2.5‐fold. In an MI mouse model, BST, with programmable biodegradation (∼3 weeks), restored left ventricular ejection fraction to ∼70% of sham group and improved 28‐day survival by ∼2.5‐fold. Furthermore, BST enables real‐time MRI/CT tracking of material retention and tissue repair dynamically, permitting spatiotemporal control of the infarct microenvironment and advancing precision MI therapy toward clinical translation.

## Graphical abstract

We developed a pH responsive theranostic serum albumin hydrogel named BST, functionalized with MRI and CT probes and loaded with mitochondria targeted CAT‐SOD nanogels, that rapidly self repairs and mechanically stabilizes the infarcted myocardium, releases therapeutic nanogels and albumin under hypoxia induced acidosis to clear mitochondrial ROS, generate oxygen, restore osmotic balance and modulate immunity, and thereby enhances cardiomyocyte survival, increases angiogenesis and cardiac function while enabling real time multimodal imaging for spatiotemporally adaptive precision therapy.

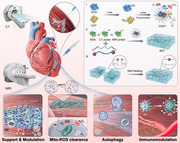

## 1 Introduction

Cardiovascular diseases remain the leading cause of death globally, with acute myocardial infarction (AMI) being the most severe outcome of heart disease [1, 2]. AMI results from sudden coronary artery occlusion, which initiates a cascade of ischemic insults including mitochondrial dysfunction, excessive reactive oxygen species (ROS) generation, anaerobic glycolysis, and lactic acidosis, ultimately leading to widespread cardiomyocyte death. This is followed by a robust inflammatory response, marked by monocyte recruitment and M1 macrophage polarization, which exacerbates tissue damage. Although fibroblast‐driven scar formation temporarily restores structural integrity, it compromises contractility and contributes to chronic ventricular stiffening and eventual heart failure [3, 4, 5, 6]. Given the multifaceted pathology of AMI, early intervention targeting the infarct microenvironment is critical. Therapeutic strategies that mitigate hypoxia and oxidative stress, modulate inflammation, and restore osmotic homeostasis during the acute phase hold great promise in preserving viable myocardium and improving long‐term outcomes. However, current clinical interventions, such as reperfusion therapy and pharmacological agents, are primarily focused on revascularization and infarct size limitation. These approaches often fall short in promoting functional regeneration and carry risks of serious complications, including bleeding [7, 8, 9, 10]. Therefore, there is an urgent need to develop therapeutic strategies that not only protect the ischemic myocardium but also actively promote multi‐stage tissue repair and functional regeneration following AMI.

Hydrogels are three‐dimensional, water‐rich networks with physical and mechanical properties similar to those of native soft tissues, and have emerged as promising platforms for myocardial repair [11]. Their inherent softness and injectability allow for minimally invasive delivery and mechanical support to the infarct zone. Furthermore, their versatility as carriers for therapeutic agents enables localized and sustained bioactivity [12, 13, 14]. Recent ROS‐regulating biomaterials, including ROS‐responsive hydrogels, immunomodulatory hydrogels, and nanozyme‐based antioxidant systems, have provided valuable strategies for remodeling pathological inflammatory or oxidative microenvironments through ROS scavenging, stimulus‐responsive therapeutic release, or enzyme‐mimetic catalytic activity [15, 16, 17]. These advances highlight the therapeutic importance of microenvironment regulation, while also suggesting that further integration of mechanical adaptability, sustained ROS/hypoxia modulation, immunomodulation, and real‐time therapeutic monitoring remains highly desirable for myocardial repair. Nevertheless, achieving these functions within a single injectable hydrogel platform while maintaining mechanical compatibility, biological responsiveness, facile administration, and translational feasibility remains challenging [18, 19, 20, 21, 22].

Protein‐based hydrogels, particularly those derived from natural biomacromolecules, offer unique advantages in terms of biocompatibility, biodegradability, and biological function [23, 24, 25, 26]. Serum albumin, a major plasma protein, is an ideal building block for multifunctional hydrogel design owing to its long in vivo half‐life, role in maintaining tissue osmotic balance, and excellent drug‐binding capacity [27]. Compared with other commonly used protein‐based materials such as collagen or silk fibroin, serum albumin offers notable advantages in terms of established clinical use, scalable industrial production, and ease of chemical modification; moreover, its intrinsic antioxidant properties and immunomodulatory functions facilitate inflammation resolution and promote repair of damaged tissues [28, 29]. Mechanistically, the redox‐active Cys34 residue of serum albumin represents a major extracellular free‐thiol reservoir and, together with methionine residues, directly scavenges reactive oxygen/nitrogen species, thereby contributing to extracellular redox homeostasis [30, 31]. In addition, albumin can bind redox‐active transition metal ions such as Cu and Fe to attenuate Fenton‐type radical generation, while its hypochlorous acid‐scavenging capacity under inflammatory conditions links oxidative buffering to macrophage antigen processing and MHC‐II‐dependent immune regulationc [32]. From a biomaterials perspective, maintaining the native conformation of albumin at material interfaces can reduce opsonization and macrophage scavenger‐receptor recognition, providing a molecular basis for its inflammation‐modulating behavior [33, 34]. Collectively, these attributes render serum albumin a highly translational biomaterials platform. Intriguingly, hypoalbuminemia is a well‐established independent risk factor for adverse outcomes in AMI patients, suggesting a pathophysiological role of albumin in post‐infarct recovery [35, 36, 37]. Engineering albumin‐based hydrogels that recapitulate its native functions and integrate therapeutic and diagnostic modalities may therefore offer a transformative strategy for MI treatment. Nevertheless, designing an albumin hydrogel that is injectable, self‐repairing, mechanically compatible with the dynamic beating heart, and responsive to the multifaceted post‐infarct microenvironment, while also enabling real‐time feedback for therapy monitoring, remains a formidable challenge. Overcoming this challenge is essential to unlock the full potential of albumin‐based hydrogels for myocardial repair and clinical translation.

**FIGURE 1:**
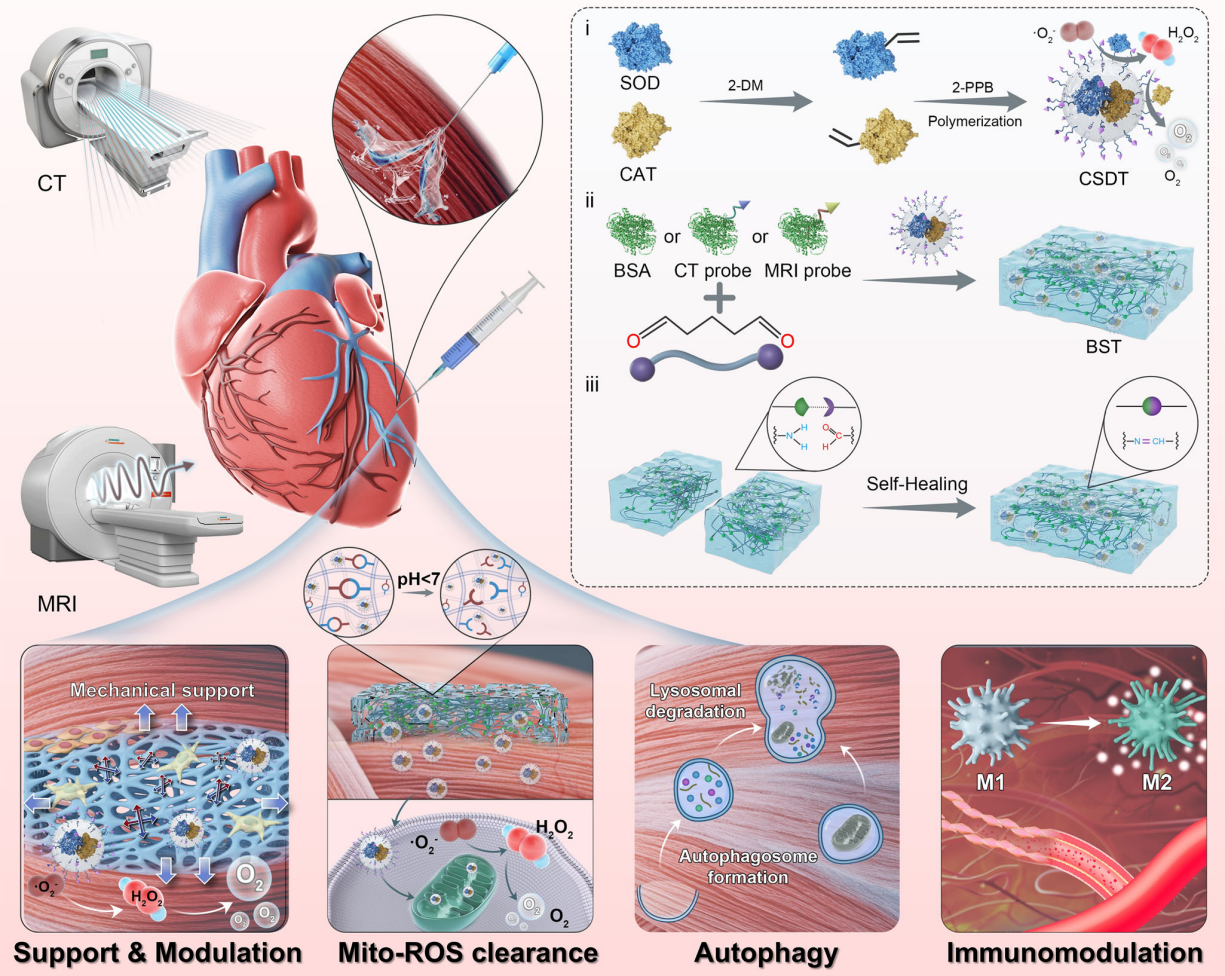
Injectable, self‐healing, and theranostic BSA hydrogel loaded with mitochondria‐targeted SOD‐CAT cascade nanogels for myocardial infarction therapy. Its tunable mechanical properties, strong adhesiveness, and rapid self‐healing capacity maintain structural stability of the infarcted region while conforming to the beating myocardium. Hypoxia‐induced acidosis triggers controlled release of composite enzyme nanoparticles and albumin from the hydrogel: the enzyme nanoparticles selectively target damaged mitochondria in cardiomyocytes, continuously scavenging mitochondria‐derived ROS and locally generating O_2_ to enhance mitophagy and anti‐apoptotic signaling; the released albumin provides antioxidant and carrier functions that help restore tissue osmotic balance and redox homeostasis, and its conjugated MRI/CT probes enable real‐time feedback on the evolving infarct microenvironment. Together, these actions alleviate local hypoxia, remodel the immune milieu by rebalancing M1/M2 macrophage polarization and attenuating acute inflammation, promote angiogenesis, and thereby drive functional repair of the infarcted myocardium.

In this study, we developed a pH‐responsive and theranostic serum albumin hydrogel (BST) functionalized with MRI/CT probes and loaded with mitochondria‐targeted CAT–SOD enzyme nanogels (CSDT) (Figure 1). The hydrogel was fabricated via dynamic Schiff base crosslinking between BSA amino groups and glutaraldehyde aldehyde groups, endowing it with high shear viscosity (∼300 Pa·s), tunable mechanical properties (Young's modulus 1∼50 kPa), and rapid self‐healing (10 min). The system enables pH‐responsive release of nanogels in the hypoxia‐induced acidic myocardial microenvironment, resulting in sustained mitochondrial ROS clearance and local O_2_ generation; these dual actions enhance mitophagy and anti‐apoptotic signaling, thereby promoting cardiomyocyte survival. This synergistically reduces oxidative stress, rebalances M1/M2 macrophage polarization, and promotes angiogenesis and cardiomyocyte survival by enhancing autophagic and anti‐apoptotic pathways. BST hydrogels degrade over three weeks, maintaining anti‐inflammatory activity to mitigate fibrosis and improving myocardial viability and functionality. Functionalization with MRI/CT agents enables real‐time bioimaging of tissue repair and drug dynamics. In AMI mouse models, BST treatment enhanced vascular density 2.5‐fold, restored ejection fraction to 70% of sham controls, and achieved 100% 28‐day survival, which represented a fivefold improvement compared to untreated groups. This multifunctional and theranostic BST protein hydrogel offers a novel and promising strategy for the treatment of acute myocardial infarction and holds strong potential for clinical translation.

## 2 Results

### 2.1 Synthesis and Characterization of BST Hydrogels

**FIGURE 2:**
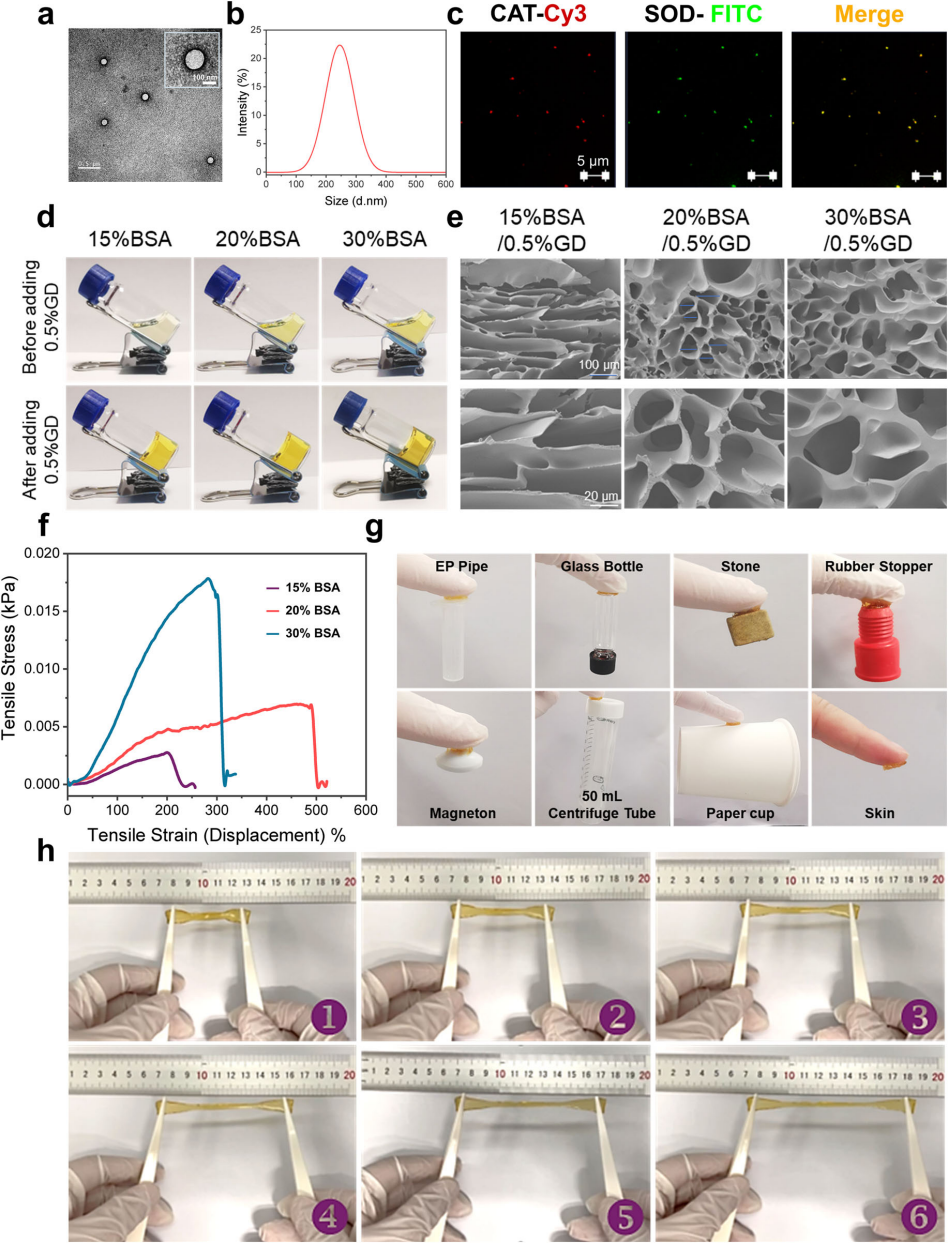
Characteristics of BST hydrogel. (a) TEM images, (b) DLS, and (c) Fluorescence colocalization analysis of CSDT; (d) Pictures of different proportions of BSA before and after gelation; (e) SEM images of different proportions of BSA after gelation; (f) Tensile properties of BSA hydrogel; (g) Adhesion of BSA protein glue to different objects; (h) Stretching pictures of BSA hydrogel.

The preparation of BST hydrogel is divided into two steps, namely the synthesis of mitochondrial targeted natural enzyme CSDT and the preparation of BSA protein gel. Referring to the previous enzyme in situ polymerization technology of the research group, we used 2‐DM to modify the double bond on the enzyme surface, and then polymerized with monomers such as acrylamide and allyltriphenylphosphonium bromide through in situ free radical polymerization to prepare CSDT natural enzyme cascade nanogel with mitochondrial targeting function. As shown in Figure 2a,b, the CSDT nanogel is spherical, with a hydrated particle size of about 240 nm and good dispersibility. By modifying CAT and SOD with Cy3 and FITC, respectively, fluorescence co‐localization analysis was performed after polymerization. As shown in Figure 2c, the red fluorescence and green fluorescence have a good overlap, indicating that the two enzymes can be effectively loaded into the same nanoparticle. Further, through fluorescence spectral analysis (Figure S1), it can be observed that the CSDT group has an obvious fluorescence resonance energy transfer phenomenon, indicating that the spatial distance between the two enzymes is very close, and further indicating that the CSDT natural enzyme nanogel is effectively synthesized. To validate the structural modification, we conducted comparative FTIR analysis of CSD before and after TPP conjugation. The spectra of CSDT (Figure S2) exhibited significant attenuation of characteristic C═C stretching vibrations from TPP at 3009 and 920 cm^−^^1^, accompanied by the emergence of a distinct P─C bond absorption peak at 1113 cm^−^^1^. These spectral changes conclusively confirm the successful synthesis of the CSDT complex through covalent conjugation.

We prepared BSA protein hydrogel by utilizing the Schiff base reaction between the amino group in BSA and glutaraldehyde. As shown in Figure 2d, after adding 0.5% glutaraldehyde, the 15%, 20%, and 30% BSA solutions all gelled, with gelation times of 10, 60, and 120 s, respectively (Figure S3a). Further SEM analysis (Figure 2e) revealed that all protein hydrogels exhibited well‐defined porous structures. With increasing BSA content, the pore size gradually decreased, yet remained within the range of 50–100 µm. FTIR spectroscopy (Figure S3b) confirmed the formation of Schiff base bonds, as evidenced by characteristic peaks at 2950 and 1650 cm^−^^1^. Furthermore, the intensity of the amide peak at 3270 cm^−^^1^ (attributed to amino groups in BSA) decreased with increasing glutaraldehyde concentration, indicating effective Schiff base linkage formation. We further evaluated the mechanical properties of the protein hydrogels with varying BSA contents, as shown in Figure S4. With increasing BSA content, both the storage modulus (G′) and loss modulus (G′′) of the hydrogels increased gradually, indicating enhanced elasticity and viscosity. Specifically, the Young's moduli of hydrogels containing 15%, 20%, and 30% BSA are approximately 1.2, 7.5, and 45 kPa, respectively (Figure S4a–d). Among them, the 20% BSA hydrogel exhibited a Young's modulus close to that of natural myocardial tissue [23]. All tested protein hydrogel formulations exhibited shear‐thinning behavior, as evidenced by a decrease in viscosity with increasing shear rate, further confirming their injectability (Figure S4e). To further assess their tensile properties (Figure 2f), we found that all hydrogels exhibited excellent stretchability, capable of being elongated to 3–6 times their original length. Among them, the 20% BSA hydrogel demonstrated the best tensile performance. We further evaluated the air burst pressure resistance of hydrogels with varying BSA concentrations (Figure S5). All protein hydrogels exhibited burst pressure resistance exceeding the normal adult blood pressure range (80–120 mmHg). The 20% and 30% BSA hydrogels showed comparable performance, both exceeding 900 mmHg, which is approximately three times higher than that of the 15% BSA hydrogel (∼300 mmHg). Based on comprehensive considerations of gelation time, injectability, tensile and rheological properties, as well as the physiological pressure range relevant to cardiac tissue, the 20% BSA hydrogel was selected for further validation. This selection is consistent with recent myocardial regeneration studies showing that mechanically compliant hydrogel scaffolds can provide a favorable biomechanical microenvironment for cardiac tissue repair and cell–matrix interactions [38, 39, 40]. We further evaluated the physicochemical properties of the 20% BSA protein hydrogel. As shown in Figure 2g, the hydrogel exhibited favorable adhesive behavior on various substrates, including paper cups, rubber, plastic, glass, and skin, indicating its potential to maintain contact with different material and tissue surfaces. The 20% BSA hydrogel also showed good stretchability and elastic recovery, as demonstrated by the tensile test in Figure 2h. To further assess its mechanical stability under repeated deformation, we performed five consecutive cyclic tensile tests (Figure S6). When the tensile strain was below 100%, the stress–strain curves showed no obvious changes during repeated loading–unloading cycles. Even at a higher strain of 140%, only slight hysteresis changes were observed, and the hydrogel maintained its structural integrity without obvious fracture or macroscopic damage. After loading CSD nanogels, the hydrogel displayed pH‐responsive release behavior, with an accelerated release rate of CSD nanogels under acidic conditions (Figure S7). Taken together, these results demonstrate that the 20% BSA hydrogel possesses favorable adhesion, mechanical adaptability, cyclic deformation tolerance, and pH‐responsive drug release capacity, supporting its potential application as an injectable and mechanically adaptable hydrogel platform for myocardial repair.

**FIGURE 3:**
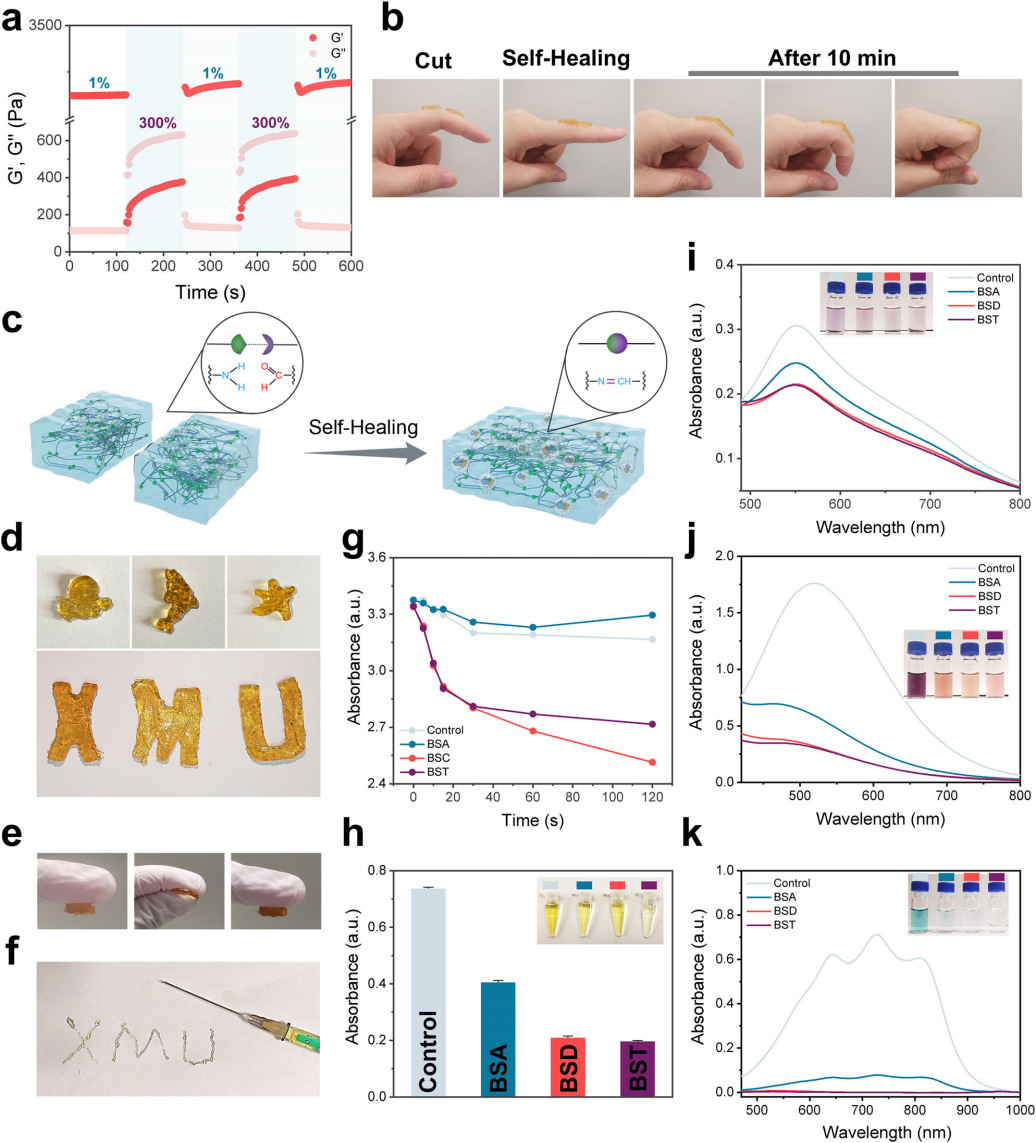
Mechanical properties of BST hydrogel and its free radical scavenging ability. (a) Rheological properties of 20% BSA protein hydrogel under three‐cycle cycling at a frequency of 1 Hz and alternating step strains from 1% to 300%. (b) Self‐healing ability of BSA hydrogel; (c) Schematic diagram of the self‐healing mechanism of BSA hydrogel; (d) Plasticity, (e) elasticity and (f) injectability of BSA hydrogel; (g) H_2_O_2_, (h)·O_2_^−^ (*n* = 3), (i) DPPH·, (j) ·OH and (k) ABTS·^+^ scavenging ability of BSA hydrogels loaded with different natural enzyme nanogels.

The self‐healing behavior of the protein hydrogel can be attributed to the dynamic Schiff base bonds formed between BSA and glutaraldehyde. As shown in Figure S8a, the storage modulus (G′) remained consistently higher than the loss modulus (G″) within the shear strain range of 0.1%–65%, indicating that the hydrogel maintained a stable gel network under moderate deformation. When the shear strain exceeded approximately 65%, G′ decreased sharply and became lower than G″, suggesting disruption of the hydrogel network under excessive shear deformation. To further evaluate the self‐healing capability of the 20% BSA hydrogel, alternating step‐strain rheological measurements were performed by switching the strain between 1% and 300% at a frequency of 1 Hz (Figure 3a). At 1% strain, G′ was higher than G″, confirming the gel‐like state of the hydrogel. In contrast, at 300% strain, G′ decreased below G″, indicating strain‐induced disruption of the hydrogel network. When the strain was returned to 1%, both G′ and G″ rapidly recovered to their initial levels, and this reversible disruption–recovery behavior was maintained over three consecutive cycles. These results demonstrate the favorable self‐healing capacity of the 20% BSA hydrogel. We also performed repeated frequency sweep measurements over an angular frequency range of 0–100 rad/s to assess the rheological stability of the hydrogel. The 20% BSA hydrogel maintained G′ higher than G″ during repeated measurements, further confirming the stability of its gel network. In addition, the shear viscosity of the hydrogel was measured repeatedly at a shear rate of 1 s^−1^ (Figure S8b). No obvious change in shear viscosity was observed during repeated testing, indicating that the 20% BSA hydrogel possesses stable viscous behavior under the tested shear condition. To further validate its self‐repair capability, we evaluated the hydrogel's recovery after being cut (Figure 3b). After 10 min, the hydrogel exhibited excellent self‐healing performance (Figure S9), maintaining its stretchability even post‐repair. The self‐healing mechanism is likely driven by the dynamic exchange between aldehyde and amine groups in the Schiff base bonds (Figure 3c). Additionally, the hydrogel demonstrated remarkable elasticity, malleability, and injectability (Figure 3d–f), making it a promising candidate for biomedical applications. Following myocardial infarction, local tissue hypoxia triggers oxidative stress and generates high levels of destructive ROS (e.g., H_2_O_2_ and ·O_2_^−^), making antioxidant capacity critical for the hydrogel [41, 42, 43]. As shown in Figure 3g,h, the CSDT‐loaded hydrogel (BST) effectively scavenges H_2_O_2_ and ·O_2_^−^, indicating that CSDT retains robust CAT and SOD activities within the BSA matrix. Moreover, BST converts H_2_O_2_ and ·O_2_^−^ to O_2_ via the SOD–CAT cascade (Figure S10), potentially alleviating myocardial hypoxia. Further evaluations using additional free radical models, including DPPH·, ·OH, and ABTS·^+^, demonstrated that although the BSA hydrogel exhibited intrinsic radical‐scavenging activity, the incorporation of CSDT nanogels markedly enhanced its antioxidant performance. Repeatability experiments further confirmed the robust and reproducible scavenging capacity of BST against multiple types of free radicals (Figure 3i–k and Figure S11). Collectively, these results indicate that the broad‐spectrum free radical‐scavenging ability of BST provides a solid basis for mitigating oxidative stress in infarcted myocardium.

### 2.2 In Vitro Antioxidant and Oxygen‐Generating Properties of BST Hydrogels

**FIGURE 4:**
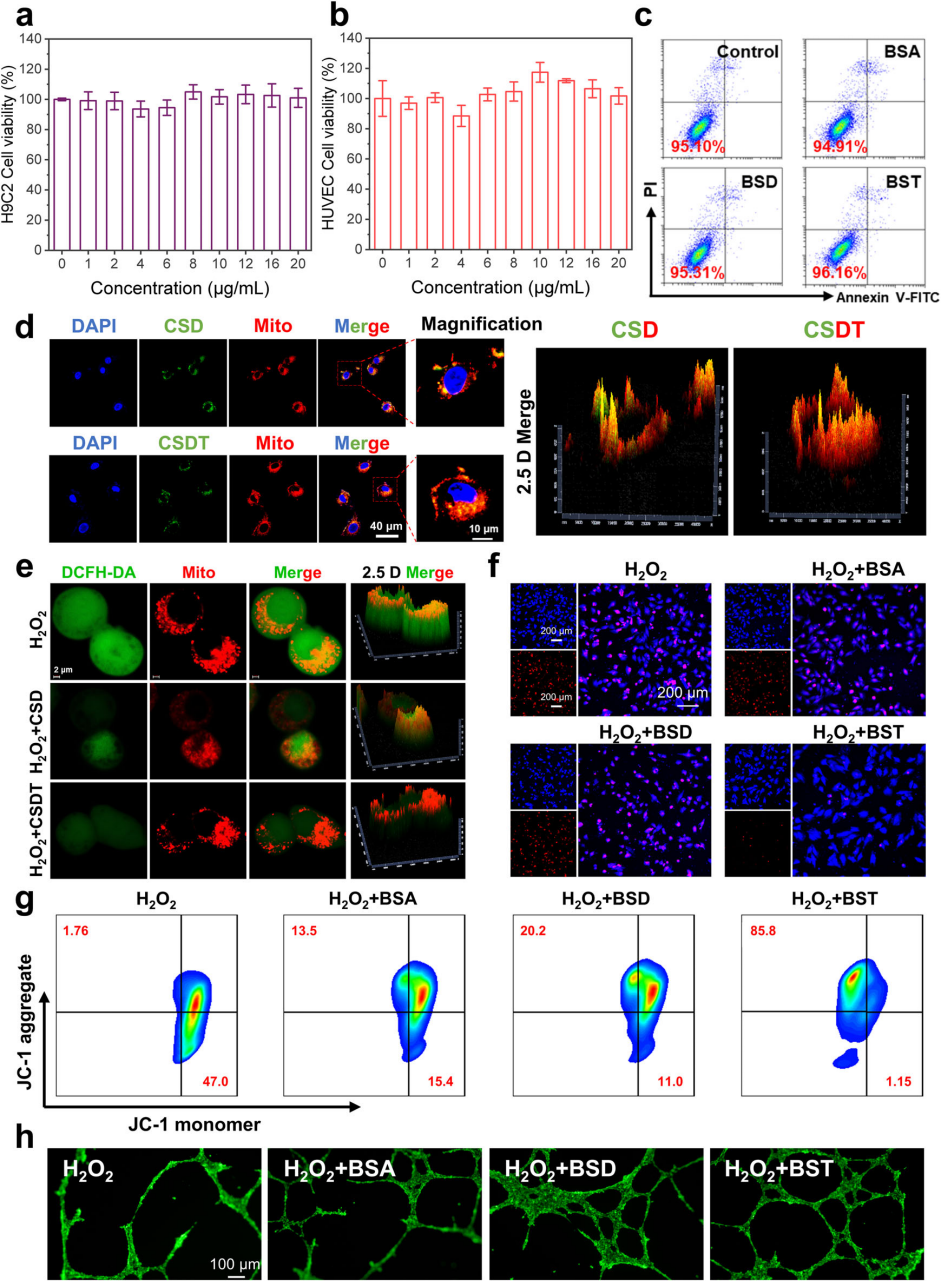
In vitro protective effect of BST hydrogel on cells with potent ROS scavenging and oxygen generation capabilities. Cytotoxicity of BST hydrogel toward (a) H9C2 cells and (b) HUVEC cells (*n* = 5). (c) Cytotoxicity of H9C2 cells treated with BSA protein hydrogel loaded with different natural enzyme nanogels was detected by flow cytometry using an Annexin V‐FITC/PI Apoptosis Detection kit; (d) CLSM and 2.5D images of H9C2 cells treated with FITC‐labeled nanogels for 8 h. Mitochondria were stained with MitoTracker Red (red fluorescence), nuclei were stained with DAPI (blue fluorescence), and nanogels (green fluorescence). Scale bar: 40 µm; (e) CLSM and 2.5D images of ROS staining of H9C2 cells treated with CSDT nanogels and H_2_O_2_ using DCFH‐DA probe, Red fluorescence represents mitochondria and green fluorescence represents ROS; (f) Fluorescence image of O_2_ staining of H9C2 cells treated with BST and H_2_O_2_ by Ru(dpp)_3_]Cl_2_ probe; (g) After co‐incubation of H9C2 cells with BSA protein hydrogels loaded with different natural enzyme nanocapsules, mitochondrial damage analysis of H9C2 cells by flow cytometry using JC‐1 probe. (h) Fluorescence images of the tube‐forming behavior of HUVEC cells after co‐incubation of HUVEC cells with BSA protein hydrogels loaded with different natural enzyme nanogels.

To further verify the biological performance of BST in vitro, we used rat cardiomyocytes H9C2 and human umbilical vein endothelial cells HUVEC as model cells for research. BST showed good biocompatibility. CSDT concentration was within 20 µg/mL, and it had good biocompatibility for both cells (Figure 4a,b, Figures S12 and S13a,c), and it also had a certain effect on promoting cell proliferation. Further flow cytometric analysis of cell apoptosis showed that CSDT did not induce obvious apoptosis, further confirming its favorable cytocompatibility (Figure 4c). To further verify the mitochondrial targeting effect of the TPP target head, we compared the targeting of natural enzyme nanogels to cardiomyocyte mitochondria in the presence or absence of TPP. The results showed (Figure 4d and Figure S14a) that CSDT in the presence of TPP had better mitochondrial targeting than CSD in the absence of TPP, as manifested by more overlap between green fluorescence (natural enzyme nanogel) and red fluorescence (mitochondria), and it could effectively remove excess ROS in cells and mitochondria through the enzyme cascade reaction of SOD and CAT (Figure 4e, Figures S14b and S15) and convert them into therapeutic O_2_ (Figure 4f), thereby preventing ROS from damaging the function and structure of mitochondria. The measurement of the membrane potential of cell mitochondria again showed (Figure 4g and Figure S16) that BST can effectively prevent ROS from damaging mitochondria and causing a decrease in their membrane potential. Moreover, the O_2_ generated by the SOD‐CAT cascade reaction not only effectively alleviates the hypoxic microenvironment (Figure S13b,d) but also promotes the tube formation of HUVEC cells (Figure 4h and Figure S17) and promotes angiogenesis. These results demonstrate that BST protein hydrogel can efficiently convert harmful ROS (e.g., H_2_O_2_ and ·O_2_^−^) into beneficial O_2_ via an enzymatic cascade, thereby potentially alleviating both oxidative stress and hypoxia in acute myocardial infarction (AMI) tissue. To elucidate BST's cytoprotective mechanism, cardiomyocytes were exposed to hypoxia and elevated ROS and assayed for the mitophagy marker LC3B (Figure S18) and the anti‐apoptotic protein BCL‐2 (Figure S19). Compared with controls, BST treatment markedly upregulated LC3B and BCL‐2, indicating enhanced mitophagy and increased anti‐apoptotic capacity. These combined effects likely act synergistically to improve cardiomyocyte survival under ischemic stress. Collectively, the data indicate that BST promotes cardiomyocyte survival and repair by scavenging mitochondrial ROS and generating local oxygen, providing a mechanistic rationale for its in vivo efficacy in a myocardial infarction model.

### 2.3 Immunomodulatory and Anti‐Inflammatory Effects of BST Hydrogel

**FIGURE 5:**
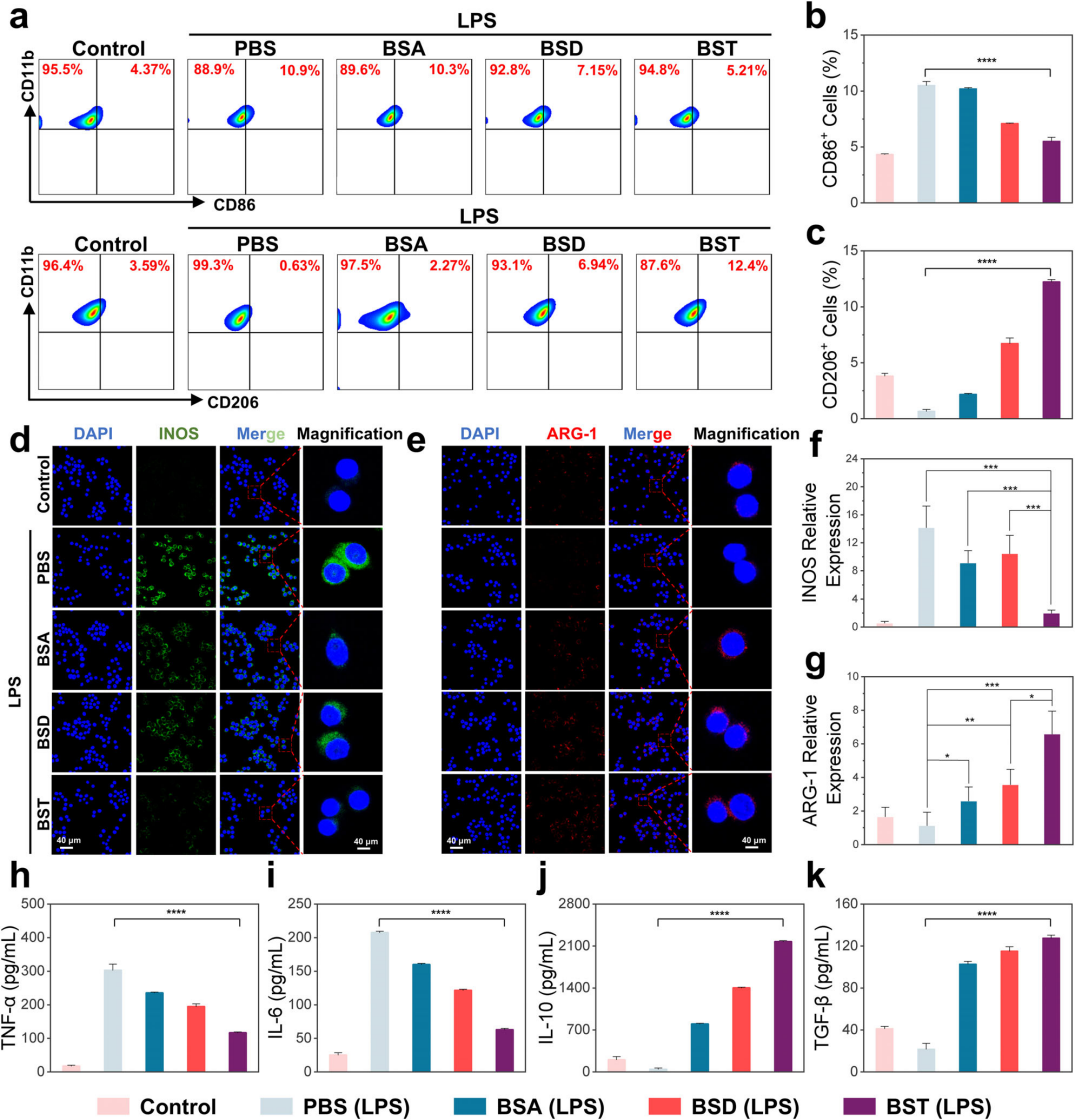
Immunomodulatory effects of BST. (a) Flow cytometric analysis of the effects of BSA protein hydrogels loaded with different natural enzyme nanogels on macrophage phenotypes; Quantitative analysis of (b) M1 (CD 86^+^) and (c) M2 (CD 206^+^) macrophage phenotypes by BSA protein hydrogels loaded with different natural enzyme nanogels (*n* = 3); Immunofluorescence staining analysis of the effects of BSA protein hydrogels loaded with different natural enzyme nanogels on the phenotypes of (d) M1 (INOS) and (e) M2 (ARG‐1) macrophages; Semi‐quantitative analysis of (f) Figure 5d and (g) Figure 5e immunofluorescence images (*n* = 3); After being treated with BSA hydrogels loaded with different natural enzyme nanogels, the release levels of different inflammatory factors secreted by macrophages (*n* = 3): (h) TNF‐α, (i) IL‐6, (j) IL‐10 and (k) TGF‐β. Data are presented as the mean ± standard deviation and were analyzed by an unpaired *t* test. ^*^ *p *≤ 0.05, ^**^ *p* ≤ 0.01, ^***^ *p *≤ 0.001, ^****^ *p* ≤ 0.0001.

Inflammation is a major factor affecting tissue damage and remodeling after myocardial infarction. Macrophages play an important role in the regulation of inflammation, but the sharp increase in ROS and increased hypoxia in myocardial infarction tissues lead to an imbalance in the number of polarized cells between the pro‐inflammatory M1 phenotype and the anti‐inflammatory M2 phenotype, significantly affecting the course of the inflammatory response [44, 45]. Reducing ROS levels and hypoxia have been shown to alleviate the inflammatory response by rebalancing the number of M1/M2 macrophages. Therefore, these results indicate the in vitro immunomodulatory potential of BST, rather than fully predicting in vivo macrophage reprogramming. We systematically evaluated the in vitro anti‐inflammatory potential of BST. As shown in Figure 5a–c, flow cytometry analysis showed that compared with the LPS group, after BST treatment, the number of pro‐inflammatory M1 phenotype (CD86^+^) macrophages was significantly reduced, and the number of anti‐inflammatory M2 phenotype (CD206^+^) macrophages was significantly increased. To further evaluate the ability of BST to rebalance the number of M1/M2 macrophages, we used INOS (M1 macrophage marker protein) and ARG‐1 (M2 macrophage marker protein) for evaluation by immunofluorescence staining. Immunofluorescence results (Figure 5d–g) showed similar results. After BST treatment of macrophages, the expression of INOS was significantly reduced, and the expression of ARG‐1 protein was significantly increased, which was manifested by decreased green fluorescence intensity and increased red fluorescence. The relevant inflammatory factors (Figure 5h–k) were measured, and it was also found that the pro‐inflammatory factors (TNF‐α and IL‐6) secreted by macrophages after BST treatment were significantly reduced, and the levels of anti‐inflammatory factors (IL‐10 and TGF‐β) were significantly increased. These results suggest that BST can regulate macrophage inflammatory responses in vitro, possibly by reducing ROS‐ and hypoxia‐related pro‐inflammatory cues [46, 47]. However, macrophage activation is highly context‐dependent, and simplified M1/M2 markers should be interpreted cautiously [48]. In addition, the LPS‐induced model represents an acute endotoxin‐driven inflammatory stimulus, which cannot fully reproduce the sterile, temporally dynamic, and multicellular inflammatory microenvironment after myocardial infarction [49]. In summary, BST has the potential to alleviate inflammatory responses in myocardial infarction tissue by rebalancing the number of M1/M2 macrophages, reducing the secretion of pro‐inflammatory factors, and increasing the levels of anti‐inflammatory factors. We will further validate its anti‐inflammatory and immunomodulatory functions in in vivo experiments.

### 2.4 Versatile Cardiac Bioimaging With BST Hydrogels

**FIGURE 6:**
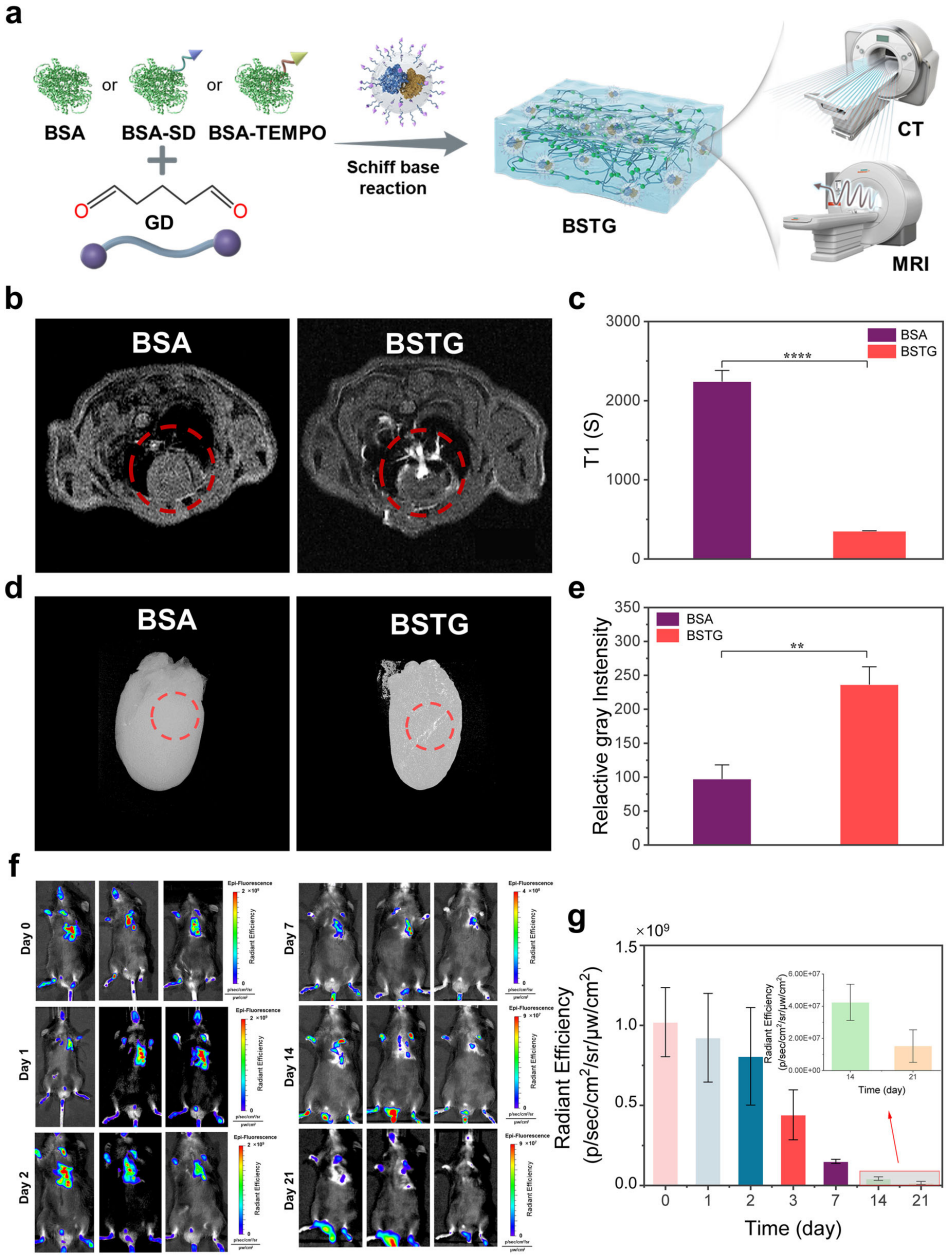
Theranostic platform based on BSA hydrogel. (a) Illustration of the preparation of BSTG protein hydrogel with CT and MRI imaging capabilities; (b) MRI images of the myocardium before and after injection of BSTG hydrogel; (c) T1 relaxation rate of BSTG in Figure S20 (*n* = 3); (d) CT images of the myocardium before and after injection of BSTG hydrogel; (e) Quantitative analysis of grayscale of BSTG hydrogel CT images in Figure S21 (*n* = 3); (f) In vivo biodistribution of RhB‐modified BST protein hydrogel after in situ myocardial injection; (g) Quantitative analysis of the fluorescence intensity of RhB‐modified BST hydrogel in the heart over time in Figure 6f (*n* = 3). Data are presented as the mean ± standard deviation and were analyzed by an unpaired *t* test. ^*^ *p *≤ 0.05, ^**^ *p* ≤ 0.01, ^***^ *p *≤ 0.001, ^****^ *p* ≤ 0.0001.

After demonstrating the antioxidant, immunomodulatory, and pro‐reparative effects of BST, we further sought to determine whether this injectable hydrogel system could be endowed with in vivo traceability. For biomaterial‐based myocardial infarction therapy, local retention of the hydrogel at the infarcted myocardium during the critical repair window is essential for sustained regulation of the post‐infarction microenvironment. Therefore, introducing imaging capability into the hydrogel system may provide a non‐invasive strategy to monitor its localization and persistence after intramyocardial injection. Bioimaging is widely used in the diagnosis, evaluation, and treatment monitoring of acute myocardial infarction (AMI). MRI provides detailed tissue and functional information for assessing myocardial viability, scar formation, inflammation, and cardiac function, whereas CT allows rapid anatomical evaluation and detection of acute cardiac complications. In this study, the MRI/CT imaging capability was incorporated not as an independent diagnostic module, but as a traceable component of the therapeutic hydrogel platform. Taking advantage of the abundant exposed amino groups on BSA, 4‐Carboxy‐TEMPO (TEMPO) and Sodium diatrizoate (SD) were conjugated to BSA through amidation reactions to obtain BSA‐TEMPO and BSA‐SD, respectively. These two functionalized BSA derivatives were subsequently crosslinked through Schiff base condensation to construct the BSTG protein hydrogel with MRI/CT dual‐modal imaging capability (Figure 6a). Compared with BSA protein hydrogel, after injection of BSTG hydrogel, the MRI signal in the heart was significantly enhanced (Figure 6b), and the T1 MRI signal was enhanced by about 8 times (Figure 6c and Figure S20). CT imaging results also showed that BSTG hydrogel can effectively enhance the CT signal of cardiac tissue (Figure 6d), and its CT signal was enhanced by about 2.5 times compared with that of BSA protein hydrogel alone (Figure 6e and Figure S21). To further observe the in vivo degradation of BST hydrogel, we further modified BSA with rhodamine B and tracked its changes over time using a small animal imaging system. As shown in Figure 6f,g, after in situ injection of BST protein gel into the myocardium, its fluorescence intensity gradually weakened over time, and its fluorescence intensity dropped sharply after 3 days, but it still had obvious fluorescence within 14 days. It was not until 21 days that the fluorescence of BST gel completely disappeared. After AMI, tissue remodeling goes through three consecutive stages: inflammation (0–3 days), proliferation and repair (about 7–10 days), and mature remodeling (>14 days to several weeks). Among them, the proliferation and repair period (especially around the 7th day) are the key time point that determines the final scar formation quality and the degree of ventricular remodeling [14, 50]. The degradation process of BST protein hydrogel coincides with the reconstruction process of acute myocardial infarction tissue, which is not only beneficial to the repair of myocardial infarction tissue but also has good biodegradability.

### 2.5 In Vivo Therapeutic Effect of BST Hydrogel on Myocardial Infarction

**FIGURE 7:**
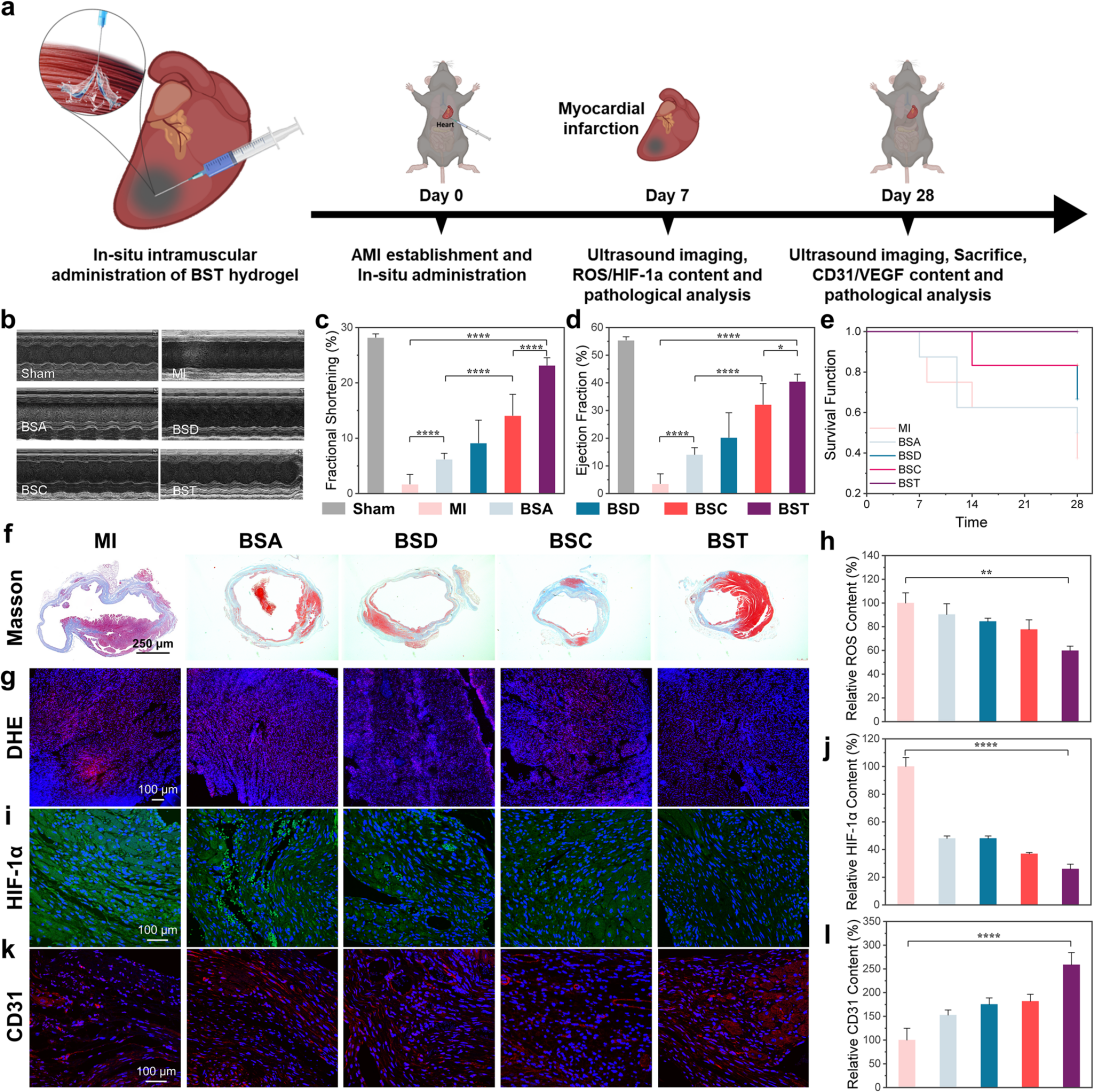
Therapeutic effect of BST protein glue on acute myocardial infarction. (a) Diagram illustrates the administration of BST hydrogel and the study of its therapeutic effect after the successful establishment of the AMI model; (b) Representative images of echocardiography on day 28; (c) Fractional shortening and (d) Ejection fraction assessed by echocardiography 28 days after BST hydrogel treatment (*n* = 5); (e) Survival curve of acute myocardial infarction mice after 28 days of treatment with different BSA protein hydrogel. (f) After 28 days of BST hydrogel treatment, mouse hearts were harvested for Masson staining analysis; After 7 days of BST hydrogel treatment, the mouse hearts were taken for (g) ROS and (i) HIF‐1α content analysis; Semi‐quantitative analysis of the fluorescence intensity of (h) ROS in Figure 7g and (j) HIF‐1α in Figure 7i (*n* = 3); After 28 days of BST hydrogel treatment, the mouse hearts were harvested for immunofluorescence staining analysis of (k) CD31 expression levels; Semi‐quantitative analysis of the fluorescence intensity of (l) CD31 in Figure 7k (*n* = 3). Data are presented as the mean ± standard deviation and were analyzed by an unpaired *t* test. ^*^ *p *≤ 0.05, ^**^ *p* ≤ 0.01, ^***^ *p *≤ 0.001, ^****^ *p* ≤ 0.0001.

The BST protein hydrogel exhibited excellent mechanical properties in vitro, including elasticity, self‐healing, and adhesiveness, along with effective capabilities for free radical scavenging and alleviation of hypoxia and inflammation. Its in vivo degradation period of approximately three weeks also helps reduce the need for repeated administration. These features render the hydrogel well‐suited to the dynamic cardiac environment and potentially beneficial for improving cardiac function. To evaluate its therapeutic efficacy, a myocardial infarction (MI) model was established in mice by ligating the left anterior descending (LAD) coronary artery, followed by in situ injection of the BST hydrogel at the time of modeling. Cardiac tissue repair was subsequently assessed at 1‐ and 4‐weeks post‐treatment using ultrasound imaging and histological analysis (Figure 7a). Echocardiography is a common method for evaluating cardiac function recovery based on left ventricular ejection fraction (LVEF). As shown in Figure 7b–d and Figure S22, the LVEF of mice in the MI group was significantly reduced compared with the Sham group, whether it was 7 days or 28 days. At 7 days, BSA treatment and BSD treatment did not significantly reverse the decrease in LVEF, but BSC treatment and BST treatment could significantly increase the LVEF of mice with myocardial infarction. This may be attributed to the dual role of SOD: although it catalyzes the dismutation of superoxide radicals (·O_2_^−^) into O_2_ and H_2_O_2_, the resulting H_2_O_2_ can further participate in reactions that generate other ROS, such as ·OH, thereby potentially exacerbating oxidative stress and myocardial tissue damage. As time progressed, the results showed that by day 28, treatment with the BSA‐based protein hydrogel significantly improved LVEF. Notably, the BST group exhibited the most pronounced recovery in cardiac function post‐myocardial infarction, with the highest average LVEF ± SED among all groups. Comparison of survival rates (Figure 7e) in mice with MI revealed a significant improvement in survival within 28 days following injection of the BST protein hydrogel. Notably, the BST‐treated group achieved an impressive 100% survival rate. This improvement may be attributed to the multifunctional properties of the BST hydrogel. Its outstanding mechanical properties enable it to adapt well to the dynamic cardiac environment and provide structural support to the infarcted myocardium. Simultaneously, the hydrogel effectively eliminates reactive oxygen species (ROS) in the infarcted tissue through an enzyme cascade reaction, while specifically targeting mitochondrial ROS and enhancing local oxygen delivery. These effects help alleviate oxidative stress, inflammatory responses, and hypoxia. Together, these synergistic actions contribute to the remodeling of the ischemic microenvironment and promote myocardial tissue repair.

To further evaluate the therapeutic effect of BST on myocardial infarction tissue, we performed histological analysis on myocardial infarction tissue. H&E staining analysis of the sections of myocardial infarction tissues of mice after 7 and 28 days of BST treatment (Figure S23) revealed that the infiltration of inflammatory cells in the myocardial infarction tissues after BST treatment was significantly reduced, and the ventricular wall was significantly thicker than that in the MI group. Myocardial fibrosis is the key to myocardial remodeling after MI and is closely related to poor prognosis. As shown by Masson staining results (Figure 7f and Figures S24, S25a,d,e), the heart volume after MI was larger, the ventricular wall was thinner, and collagen deposition was present in the left ventricle. In the BST group, the degree of cardiac fibrosis was reduced, and the thickness of the left ventricle increased. It is worth noting that BST treatment, followed by BSC treatment, had the greatest protective effect on myocardial remodeling, while BSD had the least protective effect, which is consistent with our echocardiographic results. These data reveal that BST treatment is far more effective than BSC and BSD in treating MI, which may be mainly due to its precise mitochondrial ROS scavenging effect through enzyme cascade reactions. By further performing ROS staining analysis on the heart tissue after 7 days of treatment, compared with the MI group, the myocardial tissue treated with BSA protein glue showed a significant reduction in ROS (Figure 7g,h), among which BST showed the best effect of clearing ROS. Moreover, while clearing ROS, it can also use the enzyme cascade reaction of SOD‐CAT to convert ROS (such as ·O_2_^−^ and H_2_O_2_) into therapeutic O_2_, thereby effectively reducing the expression of hypoxia‐inducible factor HIF‐1α (Figure 7i,j and Figure S25b) and alleviating hypoxia in myocardial infarction tissue. After 28 days of BST treatment, it effectively improved hypoxia, inflammatory response (such as decreased levels of pro‐inflammatory factors TNF‐a and IL‐6, increased levels of anti‐inflammatory factor IL‐10, Figure S26) and oxidative stress in myocardial infarction tissue, and promoted the expression of CD31 and VEGF in myocardial infarction tissue (Figure 7k,l, Figures S25c and S27), which is beneficial to angiogenesis in myocardial infarction tissue and promotes the repair of myocardial infarction tissue. Biocompatibility and safety were evaluated by measuring liver and kidney function markers (Figures S28 and S29), which revealed no significant differences between BST‐treated and control mice. Histological examination of major organs via H&E staining (Figure S30) likewise showed no obvious abnormalities, confirming BST hydrogel's excellent biosafety. Overall, BST is facile to fabricate and exhibits superior mechanical properties, including elasticity, viscosity, self‐healing, and extensibility, which enable it to conform to the dynamic cardiac environment and provide robust structural support to cells within the infarcted myocardium. In addition, BST demonstrates anti‐inflammatory and antioxidant activity, alleviates hypoxia, promotes angiogenesis, and possesses favorable biodegradability. These combined attributes underscore its strong potential for clinical translation in the treatment of acute myocardial infarction.

### 2.6 Mechanism of BST Hydrogel Accelerating Tissue Repair in Myocardial Infarction

**FIGURE 8:**
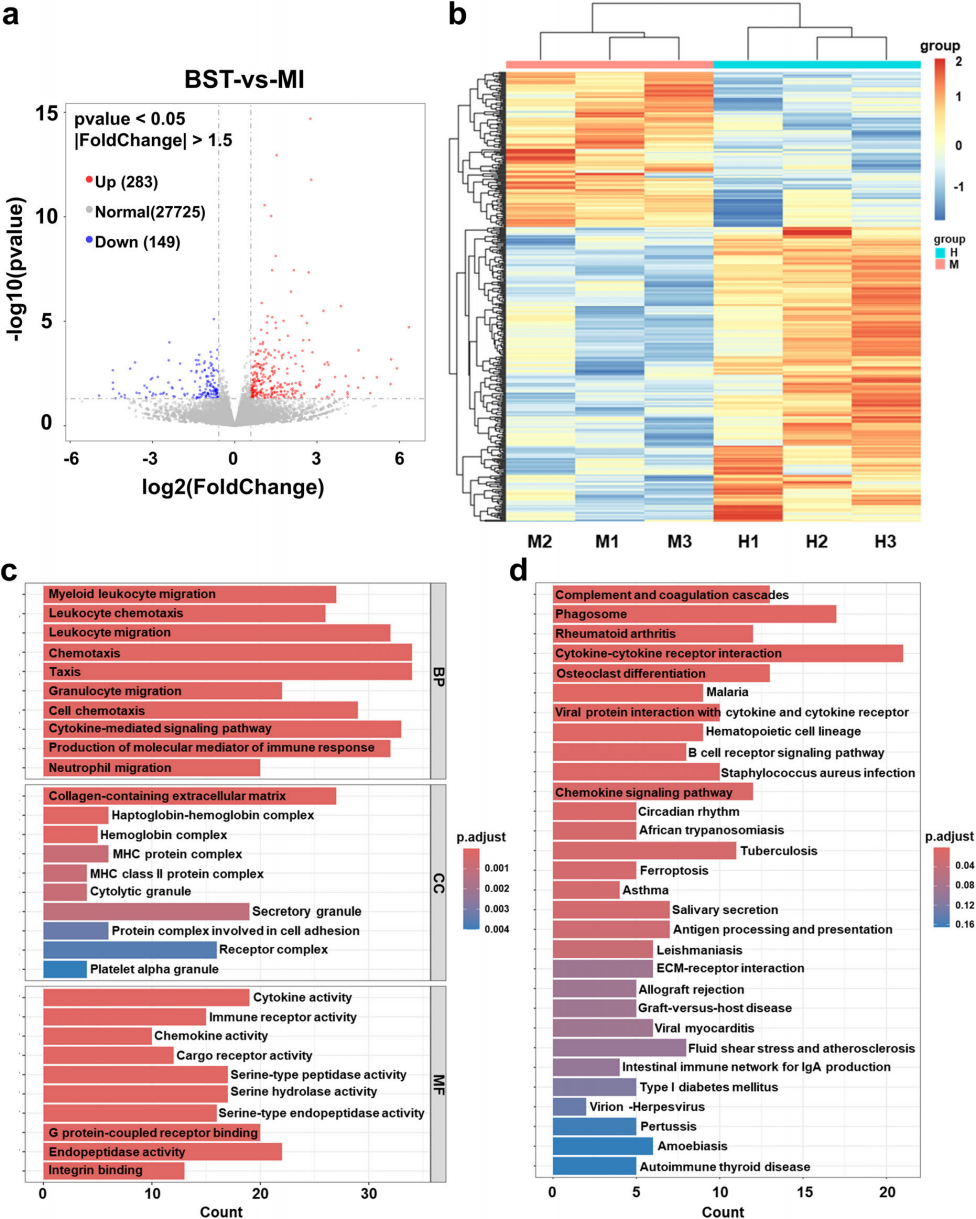
RNA sequencing of myocardial infarction tissue 28 days after BST hydrogel treatment. (a) DEGs volcano plot; (b) Hierarchical clustering heatmap of differentially expressed genes in 3 untreated and 28‐day BST‐treated MI tissues (H: BST, M: MI; n=3) (c) GO enrichment and (d) KEGG pathway enrichment analysis of differentially expressed genes.

Ischemic tissues with or without BST treatment were harvested for RNA extraction and subsequent RNA‐seq analysis. The RNA‐seq data revealed a clear separation between MI and BST‐treated samples by principal component analysis, with 432 differentially expressed genes (DEGs) (283 upregulated, 149 downregulated; Figure 8a). Hierarchical clustering confirmed that BST treatment reverses many of the MI‐induced expression changes (Figure 8b). Gene ontology (GO) enrichment analysis showed that these DEGs cluster predominantly in chemotaxis and cytokine‐mediated signaling (biological process, BP), cytokine and immune receptor activity (molecular function, MF), and collagen‐rich extracellular matrix and secretory granules (cellular component, CC) (Figure 8c). Kyoto encyclopedia of genes and genomes (KEGG) analysis further highlighted significant enrichment in cytokine–cytokine receptor interactions, autophagy, and inflammatory signaling pathways in BST‐ vs. MI‐treated hearts (Figure 8d).

Mechanistically, these transcriptomic shifts align with BST's SOD–CAT enzyme cascade: by converting ·O_2_^−^ and H_2_O_2_ into O_2_, the hydrogel attenuates ROS‐driven DAMP release and downstream recruitment of pro‐inflammatory M1 macrophages. The resulting decrease in M1‐associated transcripts and concurrent upregulation of M2 markers (e.g., IL‐10, TGF‐β) reflected in our RNA‐seq data underpin BST's immunomodulatory action. Furthermore, enhanced oxygenation may drive endothelial STAT3 phosphorylation and binding to Vegfa, Mmp2, and Pdgfb promoters, thereby increasing neovascularization in MI tissue, consistent with the observed upregulation of angiogenic pathways and our functional angiogenesis assays. Together, these data support a model in which BST remodels the ischemic microenvironment by quenching ROS, reprogramming macrophages toward an anti‐inflammatory M2 phenotype, and activating neovascular gene programs, thereby facilitating effective myocardial repair.

## 3 Conclusion

In this study, we developed a serum albumin‐based protein hydrogel (BST) integrated with mitochondria‐targeting SOD‐CAT enzyme nanogels (CSDT), designed to accelerate myocardial repair by remodeling the infarct microenvironment. The BST hydrogel exhibits excellent adhesiveness, tunable stiffness (1–60 kPa), and rapid self‐healing capability, enabling precise mimicry of myocardial mechanics and maintenance of tissue integrity in the infarcted region. The embedded CSDT is responsively released in response to hypoxia‐induced pH changes caused by lactate accumulation. Once internalized by cells, CSDT localizes to mitochondria and initiates a SOD‐CAT enzymatic cascade that scavenges excess ROS and generates O_2_, thereby dynamically regulating intracellular ROS levels and local oxygen concentration to reverse the ischemic microenvironment. This dual function rebalances M1/M2 macrophage polarization and promotes angiogenesis, contributing to effective myocardial regeneration. In an AMI mouse model, BST treatment increased vascular density by ∼2.5‐fold, restored left ventricular ejection fraction to ∼70%, and achieved a 100% survival rate, compared to only 37.5% in the untreated group. Moreover, by modifying MRI and CT probes, BST enables multimodal imaging to visualize the infarct region and monitor drug release in real time, allowing for adaptive therapeutic adjustments. Through anti‐inflammatory, antioxidant, pro‐autophagy, and oxygen‐supplementing effects, this multifunctional hydrogel effectively ameliorates the complex infarct microenvironment, offering a promising translational platform for myocardial repair in clinical AMI therapy.

## 4 Methods

### 4.1 Materials

2‐(dimethylamino)ethyl methacrylate (2‐DM), Allyltriphenylphosphonium bromide (TPP, 2‐PPB), Acrylamide (Am), Bis‐acrylamide (Bis), Hydrogen peroxide (H_2_O_2_), Tetramethylethylenediamine (TEMED), Ammonium persulfate (APS), 2,2‐Diphenyl‐1‐picrylhydrazyl (DPPH), 2,2’‐azino‐bis(3‐ethylbenzthiazoline‐6‐sulfonic acid) (ABTS), Salicylic acid (SA), Fluorescein 5(6)‐isothiocyanate (FITC), Bovine serum albumin (BSA), Glutardialdehyde (GD), Superoxide Dismutase (SOD), Catalase (CAT), SOD assay kit, 4‐Carboxy‐TEMPO (TEMPO), Sodium diatrizoate hydrate (SD) and Fetal bovine serum (FBS) was purchased from Sigma‐Aldrich. Cy3‐NHS was purchased from APExBIO. 2,2‐Azino‐bis (3‐ethylbenzothiazoline‐6‐sulfonic acid) diammonium salt (ABTS) were purchased from MACKLIN. JC‐1 probe and Phosphate buffered saline (PBS) were supplied by Solarbio Technology Co., Ltd. Antifade mounting medium with 4′,6‐diamidino‐2‐phenylindole (DAPI), Dichlorodihydrofluorescein diacetate (DCFH‐DA) were obtained from Yeasen Biotechnology Co., Ltd. tris (4,7‐diphenyl‐1,10‐phenanthroline) ruthenium (II) dichloride complex ([Ru(dpp)_3_]Cl_2_) were obtained from Bidepharm (Shanghai). iNOS antibody (CL647‐18985) and Arg‐1 antibody (CL594‐66129) were obtained from Proteintech. Bicinchoninic acid (BCA) protein assay, CD86 (MA1‐10300) and CD206 (MA5‐16872) antibodies, Mouse Endotoxin (ET) ELISA Kit (TNF‐α, IL‐6, IL‐10 and TGF‐β), DMEM (Gibco) and RPMI‐1640 (Gibco) were purchased from Thermo Fisher Scientific (USA). HIF‐1α (36169) was purchased from Cell Signaling Technology. Aspartate aminotransferase (AST) detection kits, Albumin (ALB) detection kits, Alanine aminotransferase (ALT) detection kits, and Blood urea nitrogen (BUN) detection kits were obtained from Nanjing Jiancheng Technology Co., Ltd.

### 4.2 Synthesis of CSDT Nanogel

2‐DM was added to the SOD and CAT solution (SOD:CAT = 1:1, w/w) at a molar ratio of 10 000:1 to CAT, and the mixture was stirred at room temperature for 6 h. Subsequently, TPP, Am, and Bis were added at molar ratios of 100:1, 10 000:1, and 5000:1, respectively, and the solution was stirred for 1 h under nitrogen protection. APS (APS:CAT = 550:1, mol:mol) was dissolved in 1 mL of deionized water, and TEMED (TEMED:APS = 1:1, w/w) was added to initiate the free radical polymerization on the acylated protein surface. The reaction was continued for 1 h under nitrogen protection. Finally, the Mitochondrial‐targeted CSD nanogel (TPP‐CSD, CSDT) was collected by dialysis and freeze‐drying. The synthesis of SOD nanogel and CSD nanogel was based on CSDT without adding CAT and TPP, respectively. The CSDT natural enzyme nanogels used for fluorescence colocalization analysis were synthesized by conjugating CAT and SOD with Cy3‐NHS and FITC, respectively, followed by polymerization according to the aforementioned procedures. CSD nanogels (CSD) without the mitochondrial target TPP were synthesized using the same method, except that the TPP target was missing during the synthesis process. SOD nanogels were synthesized using the same method, except that the TPP target and CAT were missing during the synthesis process.

### 4.3 Synthesis of BST Protein Hydrogel

BSA hydrogels were prepared by dissolving BSA at concentrations of 15%, 20%, and 30% (w/v) in PBS, followed by the addition of 0.5% glutaraldehyde as a crosslinker. The mixture was gently stirred and allowed to stand to form the BSA protein hydrogel. Prior to the addition of glutaraldehyde, SOD nanogels, CSD nanogels, or CSDT nanogels were sequentially added to the BSA solution to obtain BSD, BSC, and BST protein hydrogels, respectively.

### 4.4 Rheological Measurements

The rheological properties of the BST hydrogel were evaluated using a rotational rheometer (MCR 302e, Anton Paar (Shanghai) Commercial Co., Ltd.). All measurements were performed at 37°C to mimic physiological conditions. For oscillatory rheological analysis, the hydrogel sample was carefully loaded onto the rheometer platform, and excess sample was removed after lowering the measuring geometry to the preset gap. Before each test, the sample was allowed to equilibrate for several minutes to minimize mechanical disturbance.

To determine the viscoelastic behavior of the hydrogel, an oscillatory strain sweep test was first performed over a strain range of 0.01%–200% at a fixed angular frequency of 1 rad/s. The storage modulus (G′) and loss modulus (G″) were recorded as functions of strain to evaluate the linear viscoelastic region and mechanical stability of the hydrogel network.

The self‐healing behavior of the BST hydrogel was investigated by alternating low‐ and high‐strain oscillatory measurements. The hydrogel was subjected to a low strain of 1% for 120 s, followed by a high strain of 300% for 120 s. This cyclic strain protocol was repeated three times at a frequency of 1 Hz and a temperature of 37°C. Changes in G′ and G″ during the repeated strain cycles were monitored to assess the structural breakdown and recovery behavior of the hydrogel network.

To evaluate the injectability of the hydrogel, steady shear measurements were conducted at 37°C by recording viscosity as a function of shear rate. The shear rate was increased from 0.01 to 1000 s^−^^1^, and the corresponding viscosity–shear rate curve was obtained. The shear‐thinning behavior of the BST hydrogel was assessed based on the decrease in viscosity with increasing shear rate, which is indicative of its potential injectability through a syringe.

### 4.5 Tensile Testing

The tensile mechanical properties of the protein hydrogels were evaluated using a universal testing machine (68TM‐10, Instron). Dumbbell‐shaped hydrogel specimens were prepared and carefully mounted between the tensile grips. Uniaxial tensile tests were performed at a constant crosshead speed of 10 mm/min until complete fracture of the samples. The tensile force and displacement were continuously recorded during the test, and the tensile stress and elongation at break were determined from the corresponding stress–strain curves. Cyclic tensile tests were further conducted to evaluate the deformation resistance and mechanical recovery behavior of the hydrogels under repeated stretching. The specimens were stretched at the same crosshead speed of 10 mm/min, and cyclic loading–unloading tests were performed within two strain ranges: 60%–100% and 100%–140%. Five consecutive cycles were applied for each strain range, and the changes in tensile stress during repeated stretching were recorded.

### 4.6 BST Ability to Remove H_2_O_2_

The BST protein hydrogel (200 µL; 20% BSA, CSDT (20 µg/mL)) was added to a H_2_O_2_ (10 mm) PBS solution, and the rate of H_2_O_2_ consumption was determined by monitoring the absorbance at 240 nm using a UV–vis spectrophotometer.

### 4.7 Dissolved Oxygen (DO) Generation Rate Assessment

The BST protein hydrogel (200 µL; 20% BSA, CSDT (20 µg/mL)) was immersed in a 3% H_2_O_2_ PBS solution, and the dissolved oxygen (DO) levels in the solution were measured at different time intervals using a JPB‐607A portable dissolved oxygen meter.

### 4.8 BST Ability to Remove DPPH

The BST protein hydrogel (200 µL; 20% BSA, CSDT (20 µg/mL)) was mixed with an equal volume of 0.1 mm DPPH ethanol solution, followed by thorough vortexing and incubation in the dark. After 30 min, the UV–vis absorption spectrum of the solution was recorded using a UV–vis spectrophotometer.

### 4.9 BST Ability to Remove ·OH

The scavenging efficiency of BST for ·OH was measured by monitoring the content of ·OH in salicylic acid (SA). First, 0.6 mm hydrogen peroxide (H_2_O_2_) and 0.6 mm ferrous sulfate (FeSO_4_) were mixed to generate ·OH through the Fenton reaction. Then, BST protein hydrogel (200 µL; 20% BSA, CSDT (20 µg/mL)) was added to the solution to scavenge ·OH. Finally, 3 mm SA was added to detect the remaining ·OH. SA was oxidized by ·OH to form a purple‐colored 2,3‐dihydroxybenzoic acid. The absorption peak at 510 nm was further measured using UV–vis spectrophotometry.

### 4.10 BST Ability to Remove ABTS^·+^

Preparation of ABTS solution: A stock solution was prepared by dissolving 2 mm ABTS and 2.45 mm K_2_S_2_O_8_ in phosphate‐buffered saline (PBS, 25 mm, pH 7.4), followed by incubation in the dark for 4 h. Subsequently, an equal volume of ABTS^+^· solution was added to the BST protein hydrogel suspension (200 µL; 20% BSA, CSDT (20 µg/mL)). After a 30‐min reaction, the UV–vis absorption spectrum of the resulting solution was recorded using a UV–vis spectrophotometer.

### 4.11 SOD Activity of BST

The SOD enzymatic activity of the BST protein hydrogel was evaluated using a superoxide dismutase assay kit (Sigma), according to the manufacturer's instructions. The absorbance at 450 nm was measured using a microplate reader.

### 4.12 MTT Assay

The MTT assay was performed to assess the biosafety of CSDT. Briefly, H9C2 or HUVECs cells were seeded uniformly in a 96‐well plate at a density of 1 × 10^4^ cells per well and cultured overnight. CSDT was then added at a concentration gradient ranging from 0 to 20 µg/mL and incubated with the cells for 24 h. After incubation, the culture medium was aspirated, and the cells were washed three times with PBS. Subsequently, an MTT solution (final concentration: 0.5 mg/mL) was added and incubated for 4 h. The supernatant was then removed, and DMSO was added to dissolve the formazan crystals. The plate was shaken for 5 min to ensure homogeneity, and the optical density (OD) at 490 nm was measured using a microplate reader.

### 4.13 Mitochondria‐Targeting Experiment

The mitochondrial targeting capability of CSDT was verified using fluorescence imaging. Briefly, H9C2 cells (5 × 10^4^ cells/well) were seeded onto glass coverslips in a 48‐well plate and cultured overnight. The cells were then treated with FITC‐labeled CSDT and CSD for 8 h. After incubation, the supernatant was aspirated, and the cells were washed three times with PBS. Subsequently, the cells were fixed and washed again. Mitochondria were stained with Mito‐Tracker Red (1 µm) for 30 min at 37°C, followed by additional PBS washes. Finally, the coverslips were mounted with an anti‐fade mounting medium containing DAPI and visualized under a confocal laser scanning microscope (CLSM).

### 4.14 Detection of Reactive Oxygen Species (ROS)

The intracellular reactive oxygen species (ROS) levels were determined by measuring the fluorescence intensity of 2',7'‐dichlorodihydrofluorescein diacetate (DCFH‐DA) using either confocal laser scanning microscopy (CLSM) or flow cytometry. For the CLSM assay, H9C2 cells (1 × 10^5^ cells/well) were seeded evenly in confocal dishes and cultured overnight. Subsequently, the cells were treated with 80 µm H_2_O_2_ and 10 µL of the test material for 12 h. After incubation, the cells were stained with 10 µm DCFH‐DA for 30 min at 37°C in the dark. Finally, the nuclei were counterstained with Hoechst 33342, and fluorescence images were acquired using CLSM. The flow cytometry protocol followed a similar procedure, except that the cells were treated with 20 µL of the test material and nuclear staining was omitted. The fluorescence intensity of DCFH‐DA was then quantified by flow cytometry.

### 4.15 Detection of Oxygen Production Levels

The intracellular oxygen production levels were determined by measuring the fluorescence intensity of [Ru(dpp)_3_]Cl_2_ using CLSM. H9C2 cells (1 × 10^5^ cells/well) were seeded evenly in confocal dishes and cultured overnight. Subsequently, the cells were treated with 80 µm H_2_O_2_ and 10 µL of the test material for 12 h. After incubation, the cells were stained with [Ru(dpp)^3^]Cl_2_ for 30 min at 37°C in the dark. Finally, the nuclei were counterstained with Hoechst 33342, and fluorescence images were acquired using CLSM.

### 4.16 Detection of Mitochondrial Membrane Potential (MMP)in Cells

Changes in intracellular MMP (mitochondrial membrane potential) were reflected by fluorescence variations of the JC‐1 probe. Briefly, 1 × 10^5^ H9C2 cells were seeded evenly in confocal dishes and cultured overnight. Subsequently, 80 µm H_2_O_2_ and 10 µL of the test material were added to the culture medium and incubated for 12 h. After incubation, the supernatant was removed, and the cells were washed three times with PBS. The cells were then stained with 5 µg/mL JC‐1 for 30 min. Finally, MMP changes were assessed by flow cytometry, where JC‐1 aggregates (red fluorescence) indicated normal MMP, while JC‐1 monomers (green fluorescence) indicated decreased MMP.

### 4.17 Detection of Macrophage Phenotype and Cytokines

Macrophage Phenotype Validation by Flow Cytometry and CLSM. For flow cytometric analysis, 1 × 10^6^ RAW 264.7 cells were seeded evenly in 6‐well plates and cultured overnight. The cells were then treated with 100 ng/mL LPS and 20 µL of the test material for 12 h. For CD86 (M1 macrophage marker) and CD11b (pan‐macrophage marker) staining, the treated cells were collected, washed with PBS, and incubated with fluorochrome‐conjugated anti‐CD86 and anti‐CD11b antibodies for 30 min, followed by flow cytometry analysis. For CD206 (M2 macrophage marker) staining, the cells were permeabilized with cell permeabilization buffer for 15 min before incubation with anti‐CD206 antibody, and then analyzed by flow cytometry. Simultaneously, cytokine levels were quantified using commercial assay kits according to the manufacturer's protocols. Briefly, cell culture supernatants were collected from differentially treated RAW 264.7 cells and analyzed for specific cytokines (TNF‐α, IL‐6, IL‐10, and TGF‐β) using corresponding ELISA kits.

For CLSM‐based detection, 1 × 10^4^ RAW 264.7 cells were seeded onto glass coverslips in 48‐well plates and cultured overnight. The cells were treated with 100 ng/mL LPS and 20 µL of the test material for 12 h, then fixed, permeabilized, and stained withfluorescence‐labeled anti‐ARG‐1 (M2 marker) and anti‐INOS (M1 marker) antibodies. Finally, the samples were imaged using confocal microscopy.

### 4.18 In Vitro Tube Formation Assay Using HUVECs

The angiogenic potential of different treatments was evaluated using a standardized in vitro tube formation assay. Briefly, 50 µL of Matrigel was added to each well of a 48‐well plate and allowed to polymerize at 37°C for 30 min. HUVECs were then seeded onto the Matrigel‐coated wells at a density of 1 × 10^4^ cells per well. To establish an oxidative stress microenvironment, cells in the experimental groups were treated with 80 µm H_2_O_2_, followed by the addition of the corresponding test materials. After incubation, live‐cell staining was performed, and tube‐like structures were observed and imaged using fluorescence microscopy. Quantitative analysis of tube formation was conducted using ImageJ software with the Angiogenesis Analyzer plugin, including total tube length and the number of branching points.

### 4.19 Establishment of the Murine Myocardial Infarction Model

Male C57BL/6J mice (6–8 weeks old, 20–23 g) were used to induce myocardial infarction (MI). The experiments were approved by the Animal Management and Ethics Committee of Xiamen University (Animal experiment ethical review number: XMULAC20230118). Animals were anesthetized with an intraperitoneal injection of pentobarbital sodium (50 mg/kg), placed supine on a heating pad, and secured. After endotracheal intubation, mice were mechanically ventilated (tidal volume 0.2 mL, 120 breaths/min), and continuous electrocardiogram (ECG) monitoring was initiated. The chest wall was shaved and sterilized with 70% ethanol and povidone–iodine. A left thoracotomy was performed through the third or fourth intercostal space. Under a surgical microscope, the left anterior descending coronary artery (LAD) was visually identified and permanently ligated with an 8‐0 nylon suture approximately 1.5 mm distal to the tip of the left auricle. Successful occlusion was confirmed by immediate ST‐segment elevation on ECG and pallor of the anterior left ventricular wall with diminished regional myocardial contraction. The thoracic cavity was then gently evacuated of air, and the chest was closed in layers. For the sham‐operated group, the same surgical procedure was performed (with intubation, thoracotomy, and LAD exposure) except that the suture was passed beneath the LAD without being tied. All animals received postoperative analgesia and were monitored until full recovery.

### 4.20 Intramyocardial Injection

Upon confirmation of successful MI induction, intramyocardial injections were performed under a surgical microscope. Three delivery sites (one at the infarct core and two at opposing border zones) were evenly spaced within the affected myocardium. At each site, 50 µL of the hydrogel formulation was slowly injected using a 31‐gauge Hamilton syringe, taking care to avoid backflow or leakage. After all injections, the needle was left in place for 10 s before gentle withdrawal to promote gel retention. The chest was then closed as described above, and animals were recovered under standard postoperative care.

### 4.21 Cardiac Function Assessment

Cardiac function was evaluated at 1 and 4 weeks after MI surgery by transthoracic echocardiography. Mice were anesthetized with 1%–2% isoflurane in oxygen and placed supine on a heated platform to maintain body temperature at 37°C. A high‐frequency ultrasound probe was positioned immediately to the left of the sternum to obtain parasternal long‐ and short‐axis views of the left ventricle. M‐mode images were recorded at the mid‐papillary level, and left ventricular ejection fraction (LVEF) and fractional shortening (LVFS) were calculated using standard formulas to quantify systolic performance. Continuous heart rate monitoring ensured that measurements were made under stable anesthetic conditions.

### 4.22 RNA‐Seq Analysis

Heart tissues from the MI mice treated with or without BST were collected, and RNA was isolated for RNA‐seq analysis (*n* = 3).

### 4.23 Histological Analysis

The ventricle below the ligature and at the level of the papillary muscles was removed and subjected to Masson or H&E staining for tissue morphology and pathological analysis.

### 4.24 Statistical Analysis

Values are shown as mean ± standard deviation, a GraphPad Prism 9.0.0 was applied for statistical analysis. Unpaired *t*‐test was used for statistical analysis to compare statistical significance. ns: *p* ≥ 0.05, ^*^ *p* < 0.05, ^**^ *p* < 0.01, ^***^ *p* < 0.001, and ^****^ *p* < 0.0001 were respectively recognized as statistically significant, highly significant, and very significant.

## Author Contributions

Z. L. conceived the study and designed the experiments. Z. L., C. Y., L. M., K.Y. T., P. M., C. J., X.J. L., and Y.L. W. performed the experiments or provided essential experimental resources. All the authors analyzed the data. Z. L., C. Y., and L. M. wrote the manuscript with help from all the authors.

## Conflicts of Interest

The authors declare no conflicts of interest.

## Supporting information




**Supporting File**: advs76312‐sup‐0001‐SuppMat.docx.

## Data Availability

The data that support the findings of this study are available from the corresponding author upon reasonable request.
