# Supplementary material for: A Pathology‐Instructed Theranostic Platform with Mechanoadaptive and ROS‐Powered Nanobreathing Functions for Precision Myocardial Repair
Source: Adv Sci (Weinh). 2026 Jul 2:e76312. Online ahead of print. doi: 10.1002/advs.76312 (PMC13336429; doi:10.1002/advs.76312)
Supplement: Supplementary file 1 — Supporting File: advs76312‐sup‐0001‐SuppMat.docx. [file ADVS-9999-e76312-s001.docx]

Supplementary Materials for

**A Pathology-Instructed Theranostic Platform with Mechanoadaptive and ROS-Powered Nanobreathing Functions for Precision Myocardial Repair**

Zheng Luo^1,2,3^, Cui Yang^4^, Liuzhou Mao^2^, Panqin Ma^1,*^, Chiyu Jia^1,*^, Karen Yuanting Tang^3^, Xian Jun Loh^3,*^, Yun-Long Wu^2,^^*^

^1^ Center of Burn & Plastic and Wound Healing Surgery, The First Affiliated Hospital of University of South China, Hengyang Medical School, University of South China, Hengyang, Hunan 421001, China.

^2^ State Key Laboratory of Vaccines for Infectious Diseases, Xiang An Biomedicine Laboratory, Fujian Provincial Key Laboratory of Innovative Drug Target Research, School of Pharmaceutical Sciences, Faculty of Medicine and Life Sciences, Xiamen University, Xiamen, Fujian, China.

^3^ Institute of Materials Research and Engineering (IMRE), Agency for Science, Technology and Research (A*STAR), 2 Fusionopolis Way, Innovis #08-03, Singapore 138634, Republic of Singapore.

^4^ School of Medicine, Xiamen University, Xiamen 361102, China.

*Correspondence to: 2025010021@usc.edu.cn (P. Ma); 2023010003@usc.edu.cn (C. Jia); lohxj@a-star.edu.sg (X.J. Loh); wuyl@xmu.edu.cn (Y.-L. Wu).

**Figure S1**. Fluorescence emission spectra of the Cy3-CAT/FITC-SOD physical mixture (Blue Line) and CSDT natural enzyme nanogels (Red Line) under excitation at 480 nm.


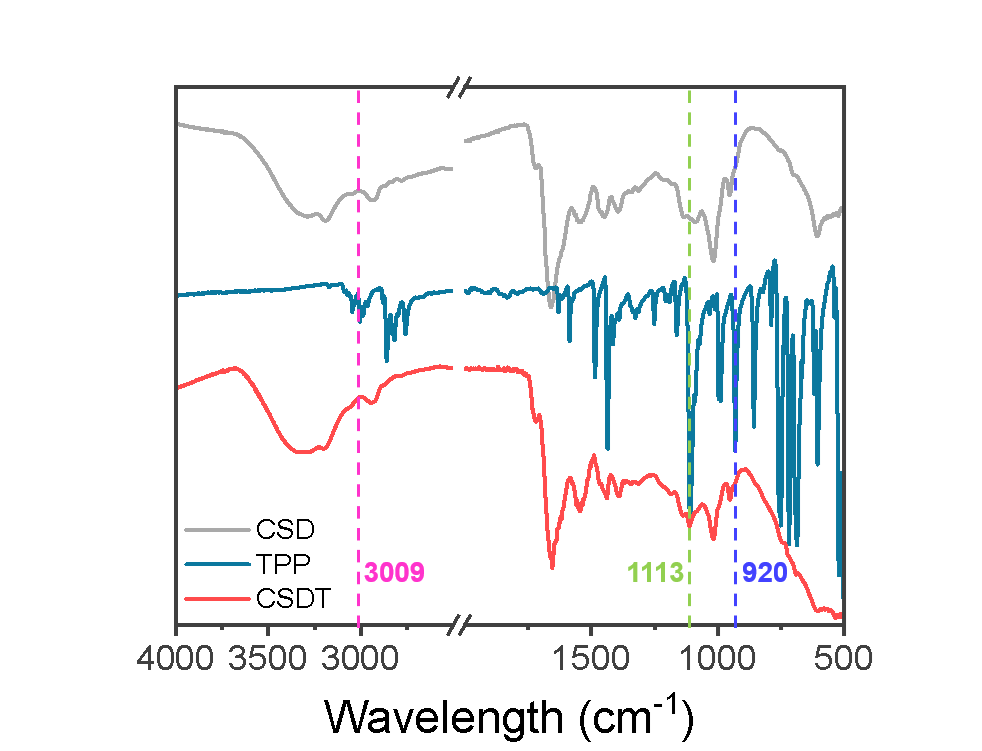


**Figure S2**. FTIR spectra of TCSD natural enzyme nanogels.


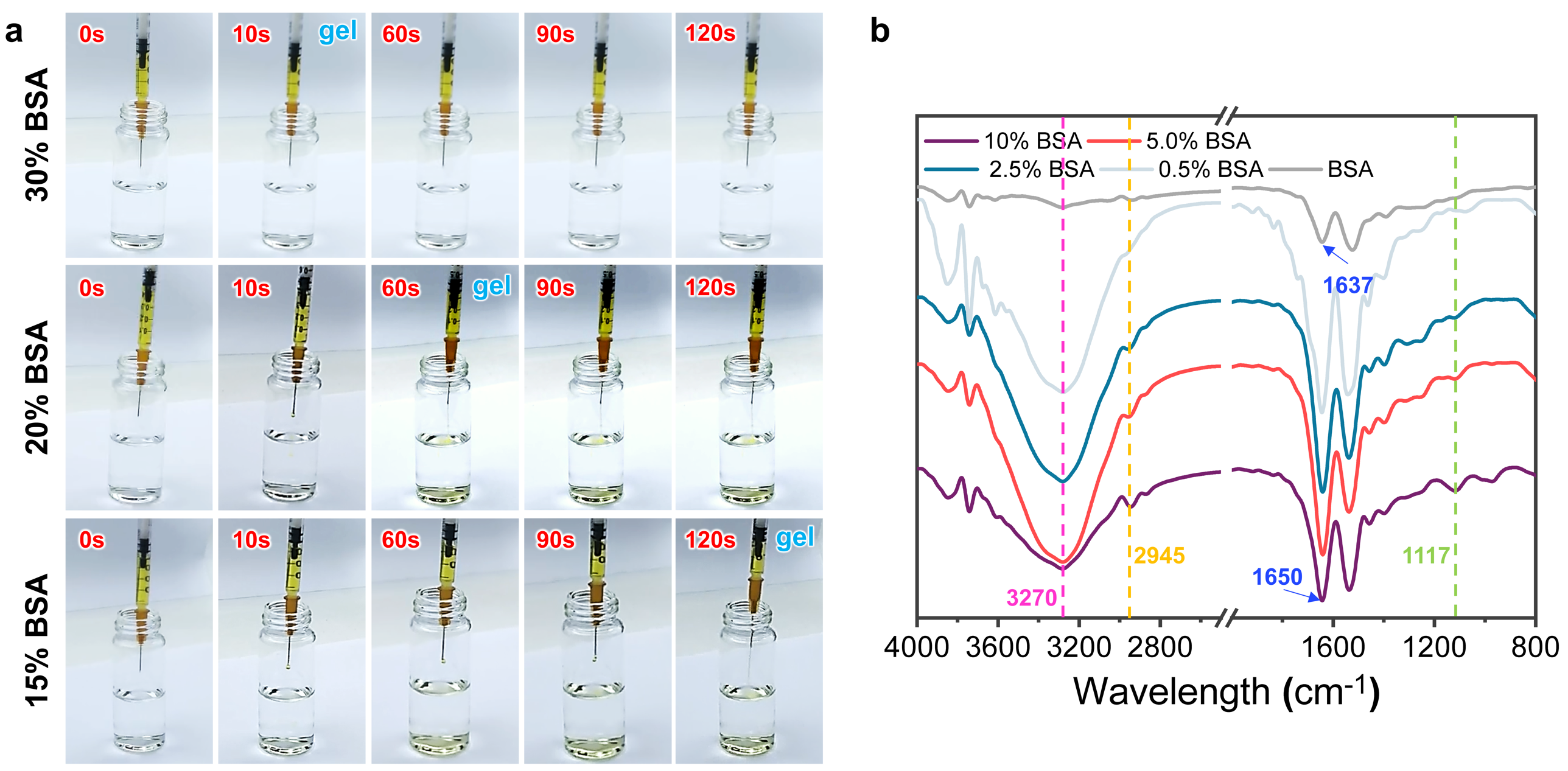


**Figure S3**. (a) Evaluation of the injectability of protein hydrogels with different proportions at different time points. (b) FTIR spectra of protein hydrogels with different BSA contents.





**Figure S4**. Frequency dependency of the storage modulus (G′) and loss modulus (G′′) at a constant shear strain of 1%: (a) 15% BSA; (b) 20% BSA and (c) 30% BSA. (d) The classical equation relating Young's modulus and storage modulus. According to the classical relation (1), Young's modulus can be estimated from the shear modulus. Since hydrogels are generally considered nearly incompressible soft materials, ϑ was taken as approximately 0.5. Therefore, the equation can be simplified to (2). To estimate the apparent Young’s modulus under a low-frequency dynamic condition relevant to cardiac tissue deformation, we used the storage modulus at an angular frequency of approximately 6.28 rad/s, corresponding to approximately 1 Hz. Based on this calculation, the apparent Young’s moduli of the 15%, 20%, and 30% BSA hydrogels were approximately 1.2 kPa, 7.5 kPa, and 45 kPa, respectively. (e) Viscosity-shear rate curve of different BSA protein hydrogel.


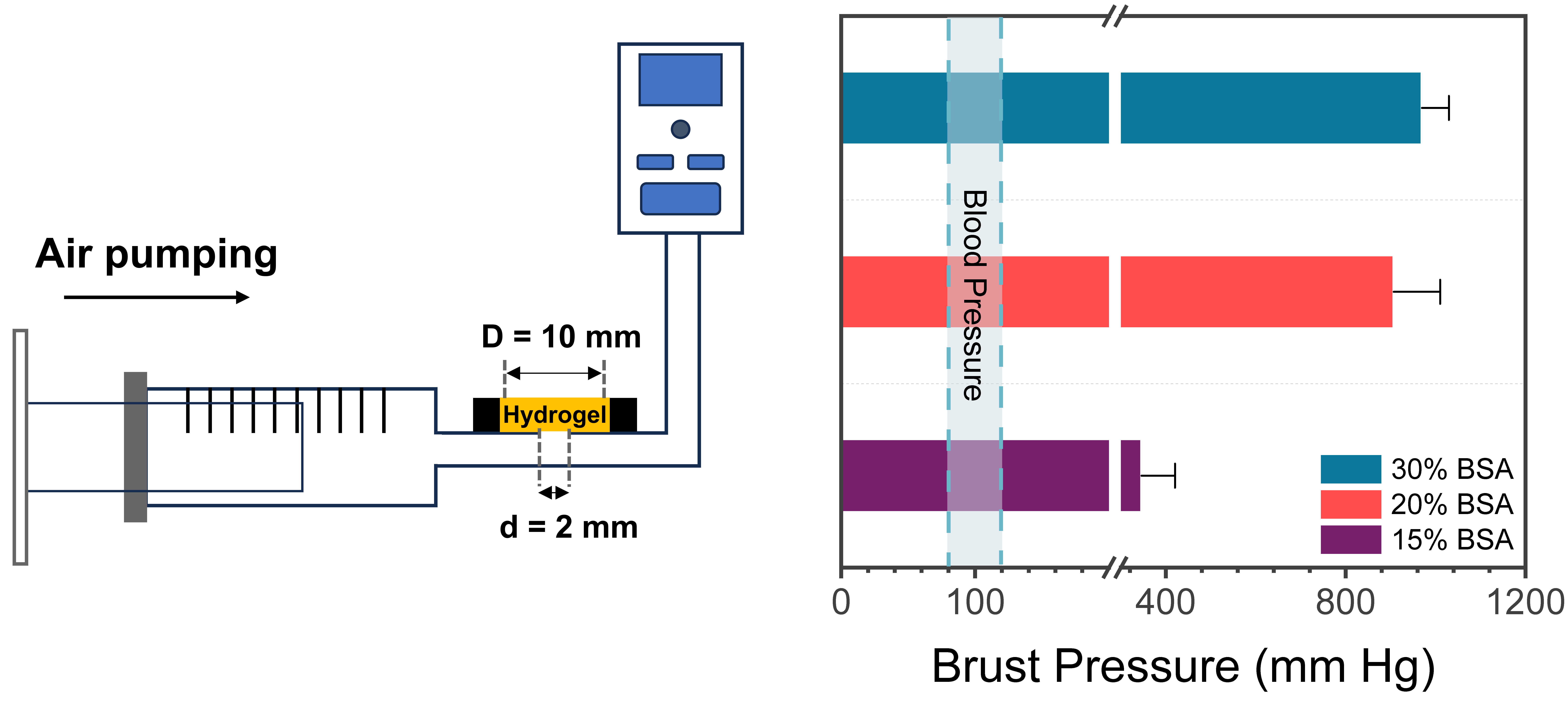


**Figure S5**. Burst pressure resistance of protein hydrogels with different proportions (n=3).


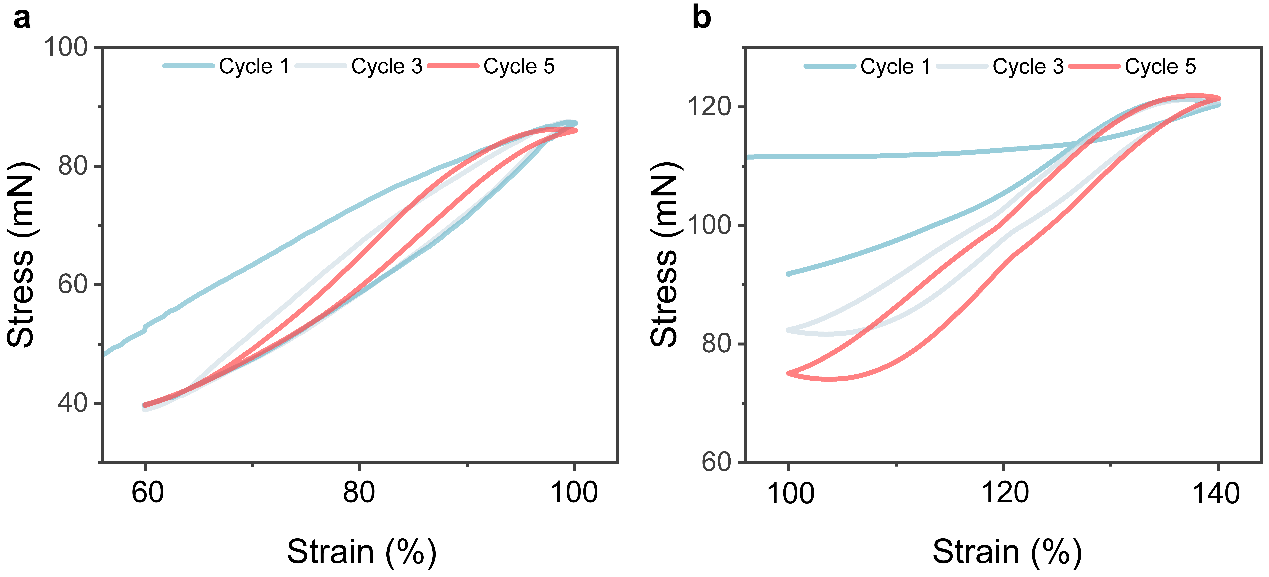


**Figure S6**. Cyclic tensile stress-strain curves of 20% BSA protein hydrogel: (f) 60%-100% strain and (g) 100%-120% strain.


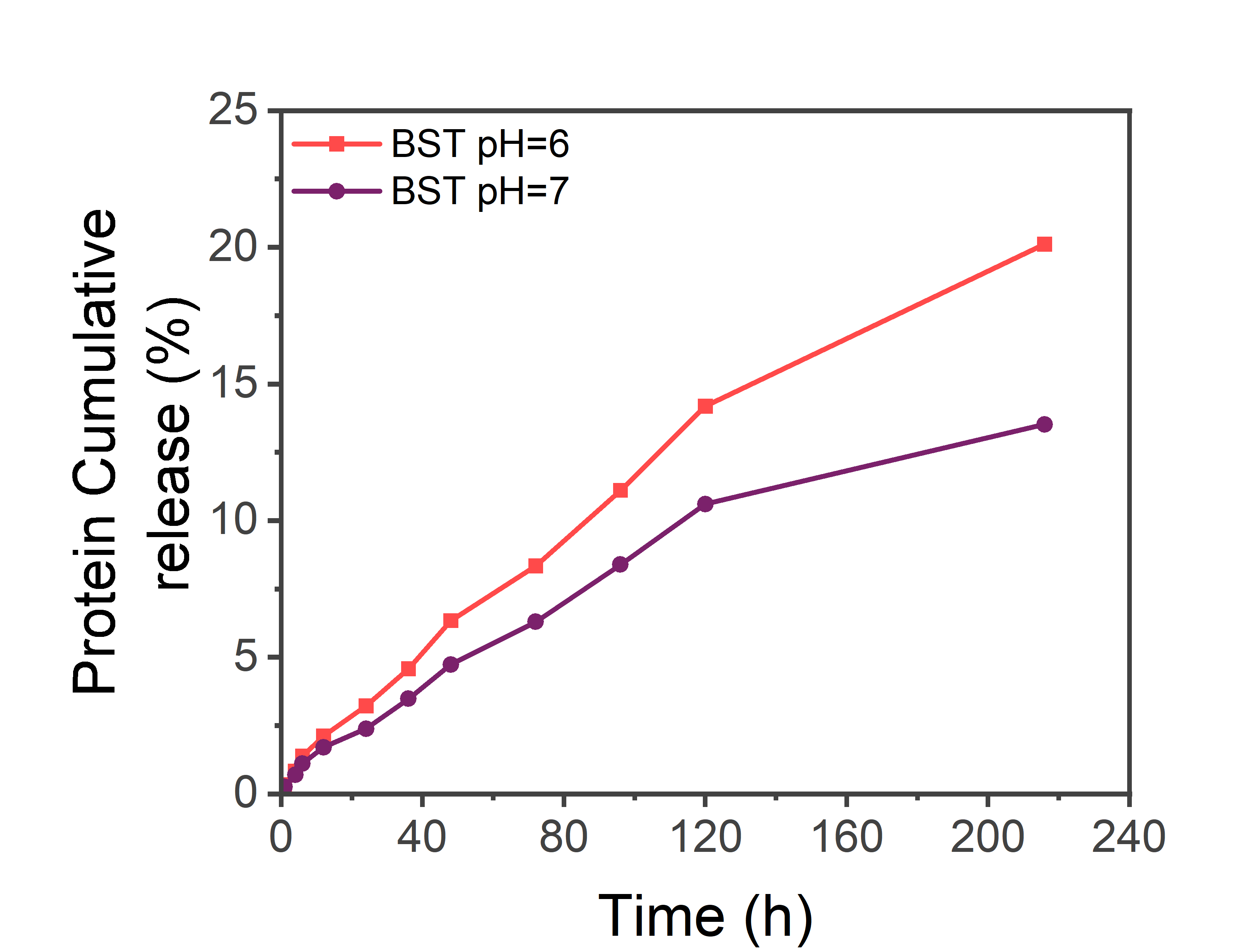


**Figure S7**. Cumulative release curves of CSD natural enzyme nanogel from BST hydrogel under different pH conditions.


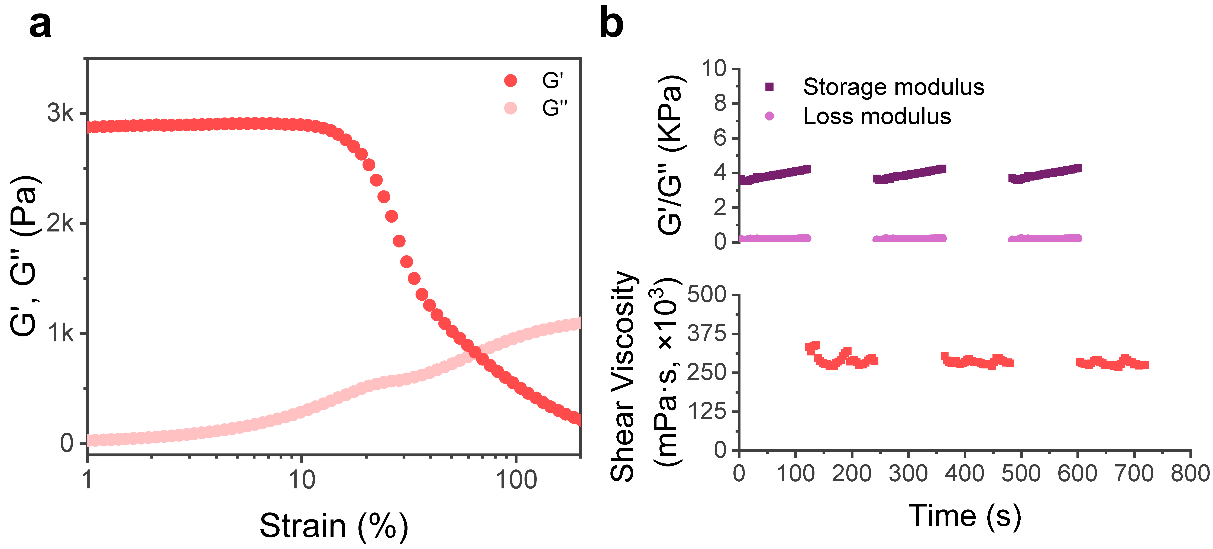


**Figure S8.** (a) Storage modulus (G') and loss modulus (G″) of a 20% protein hydrogel as a function of strain. (b) Self-healing properties of BSA hydrogels were observed in a series of continuous damage-recovery measurements. G′, G″ and viscosity was measured with increasing angular frequency (0-100 rad/s) and shear viscosity was measured under 1 s^-1^ shear rate (n = 3).


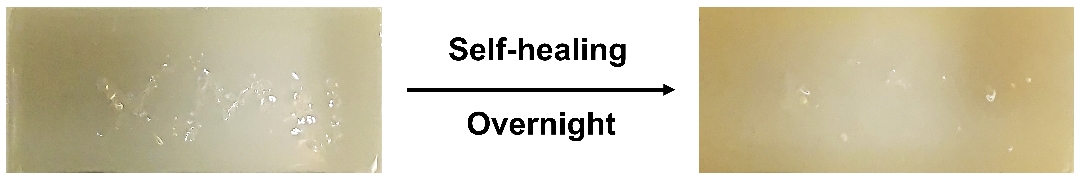


**Figure S9**. Self-healing ability of 20% BSA protein hydrogel.


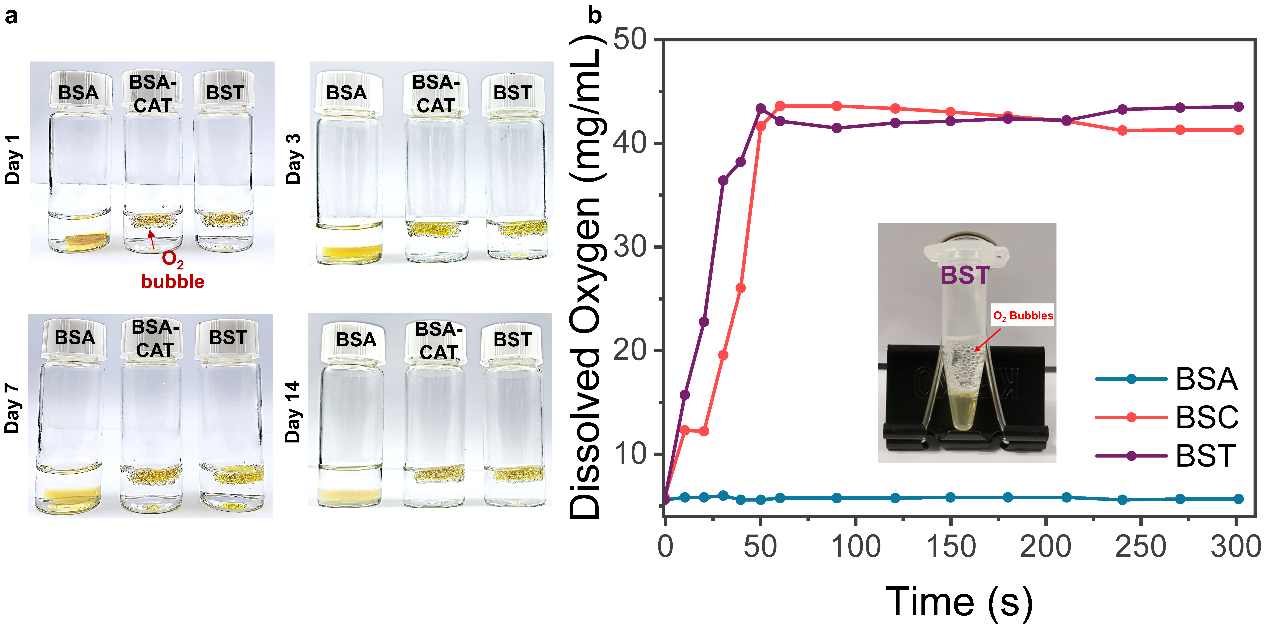


**Figure S10**. (a) BST protein hydrogel catalysis of H_2_O_2_ to O_2_ production at different time points. (b) The curve of the amount of O_2_ produced by BSA protein gel loaded with different nanogels in 3% H_2_O_2_ solution over time.


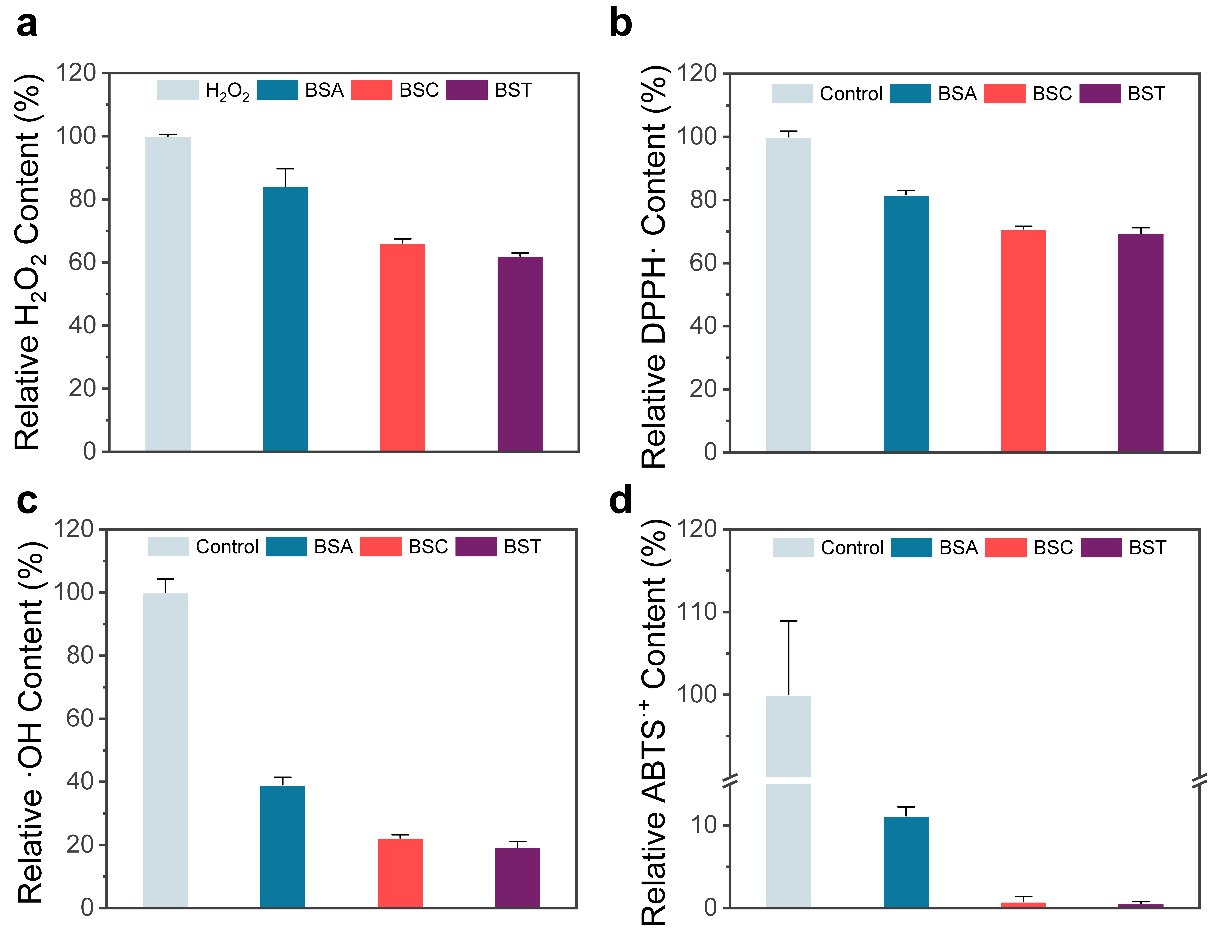


**Figure S11**. The scavenging abilities of different protein hydrogels for different free radicals (n=3) : (a) H_2_O_2_, (b) DPPH·, (c) ·OH and (d) ABTS^·+^.





**Figure S12**. (a) Cell compatibility assessment using extracts from the crosslinked BSA protein hydrogel collected at different time points. (b) Live/dead staining images of H9C2 or HUVEC cells treated with BSA protein hydrogel loaded with different natural enzyme nanogels.


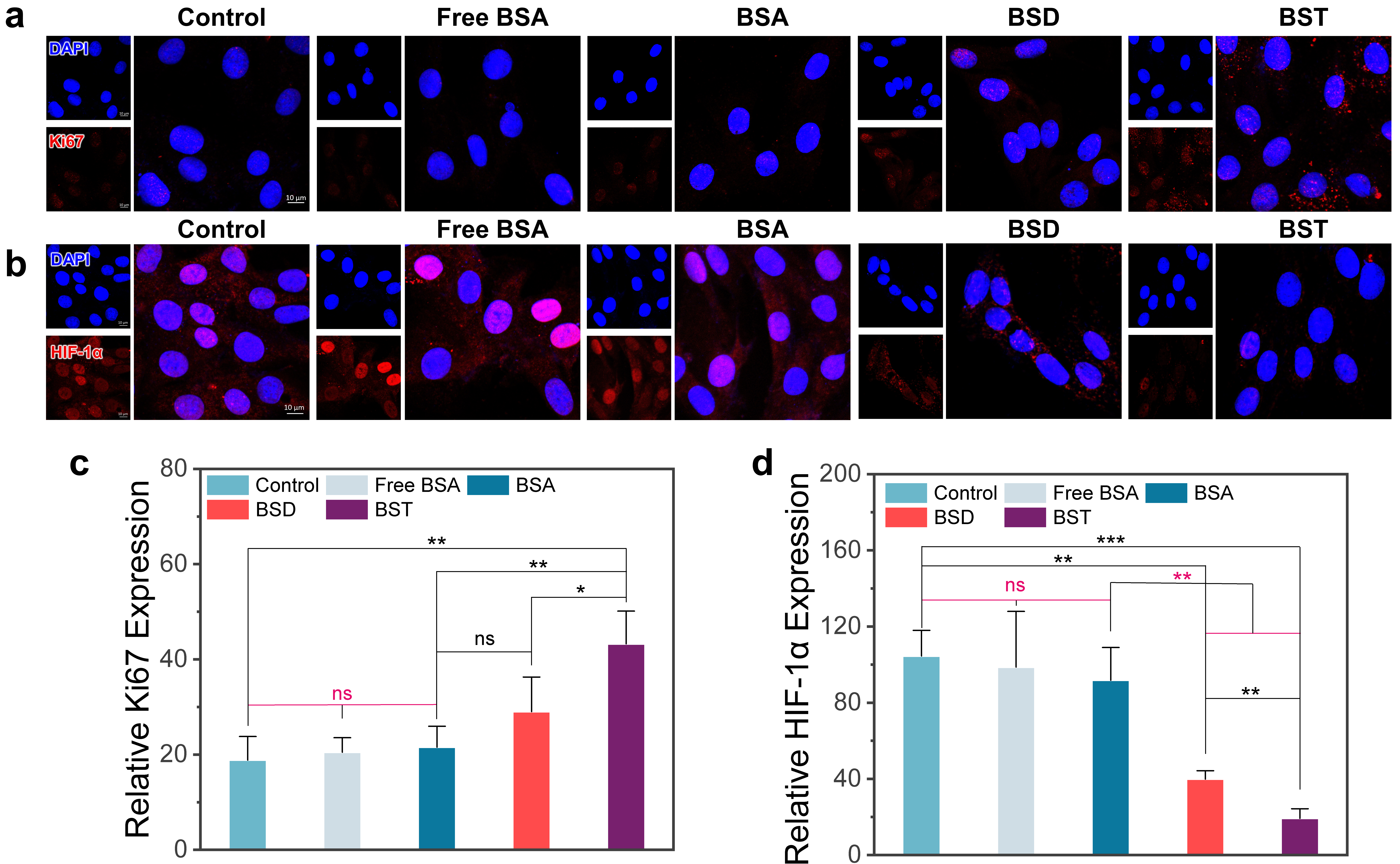


**Figure S13**. Immunofluorescence staining images showing (a) Ki67 and (b) HIF‑1α expression in H9C2 cells under hypoxic conditions with 80 μM H_2_O_2_. Semi-quantitative analysis of the fluorescence images in Fig S13a-b: (c) Ki67 and (d) HIF-1α.


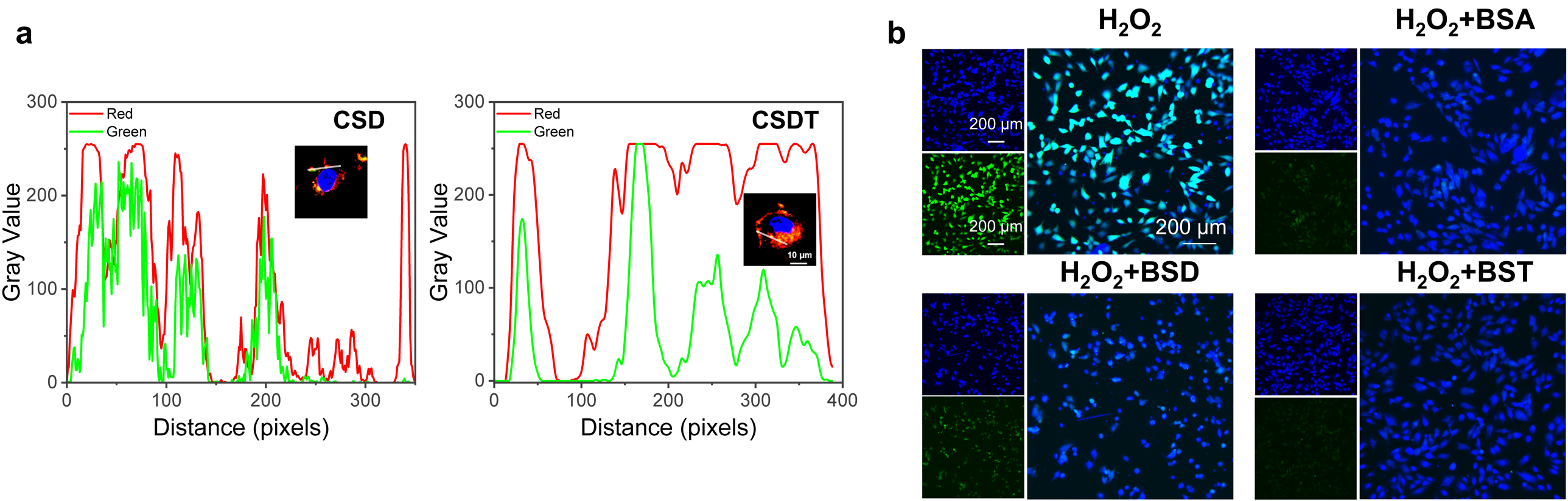


**Figure S14**. (a) Fluorescence colocalization analysis of CSDT and CSD nanogels in Figure 4D. (b) Fluorescence image of ROS staining of H9C2 cells treated with CSD nanogel and H_2_O_2_ by DCFH-DA probe.


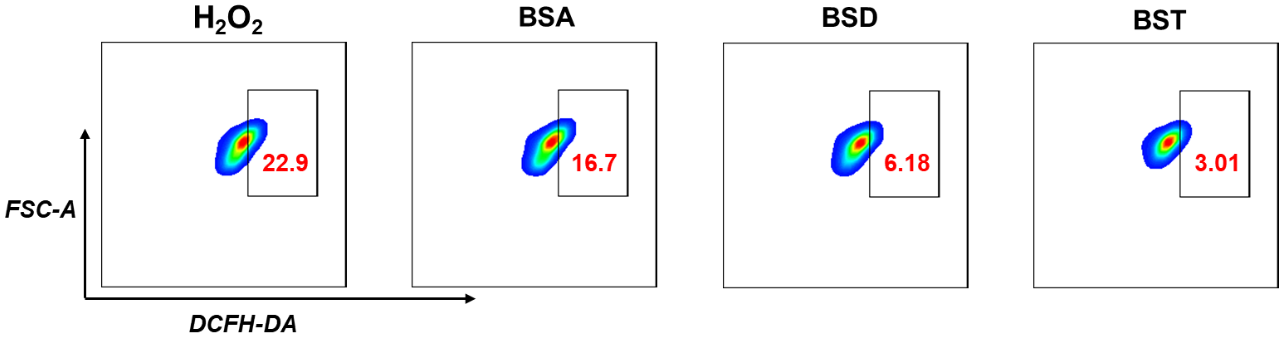


**Figure S15**. Live/dead staining images of HUVEC cells treated with BSA protein hydrogel loaded with different natural enzyme nanogels.


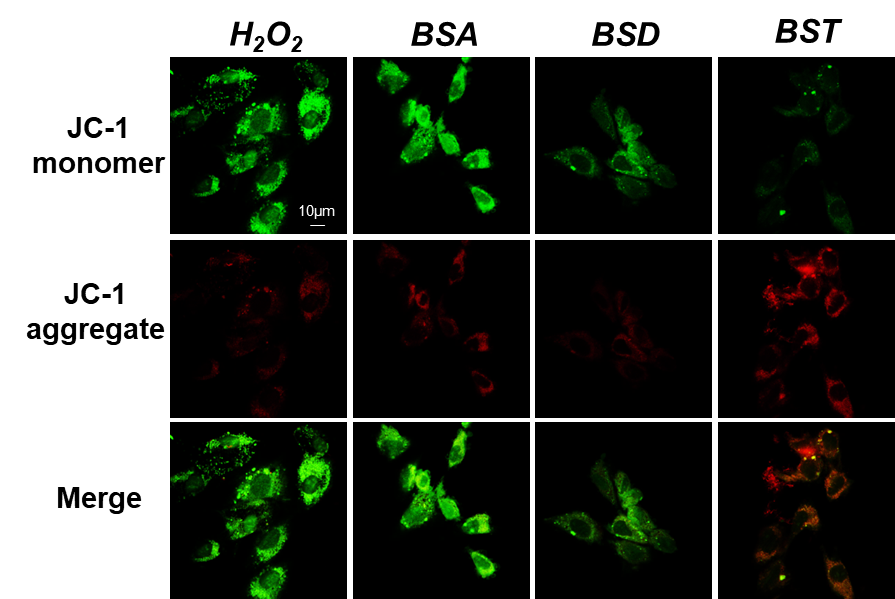


**Figure S16**. After H9C2 cells were treated with 80 μM H_2_O_2_ and loaded with different natural enzyme nanogels, their mitochondrial membrane potential was analyzed using the JC-1 probe.


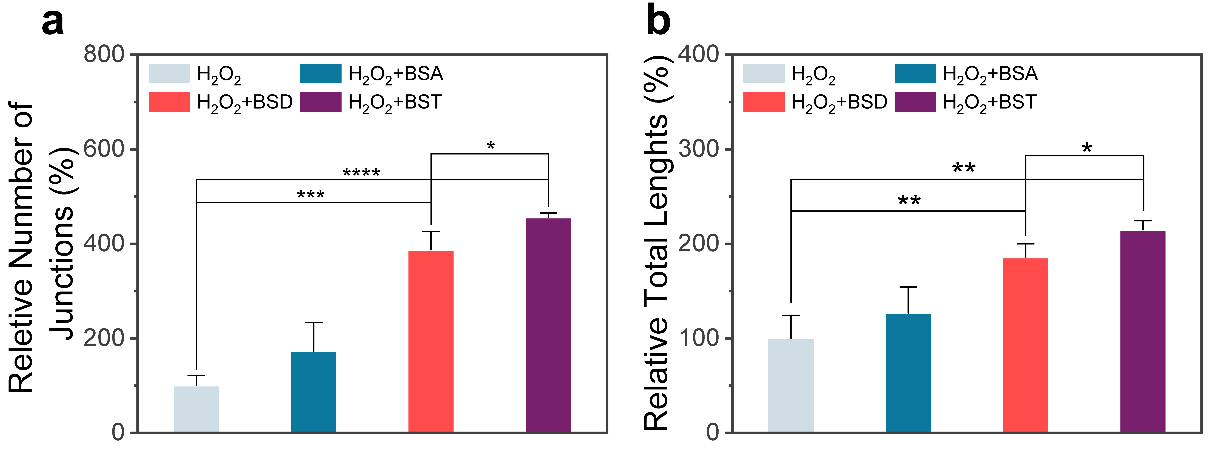


**Figure S17**. ImageJ quantification of HUVEC tube formation in Fig. 4h: (a) relative number of junctions and (b) relative tube length after treatment with different protein hydrogels (n=3).


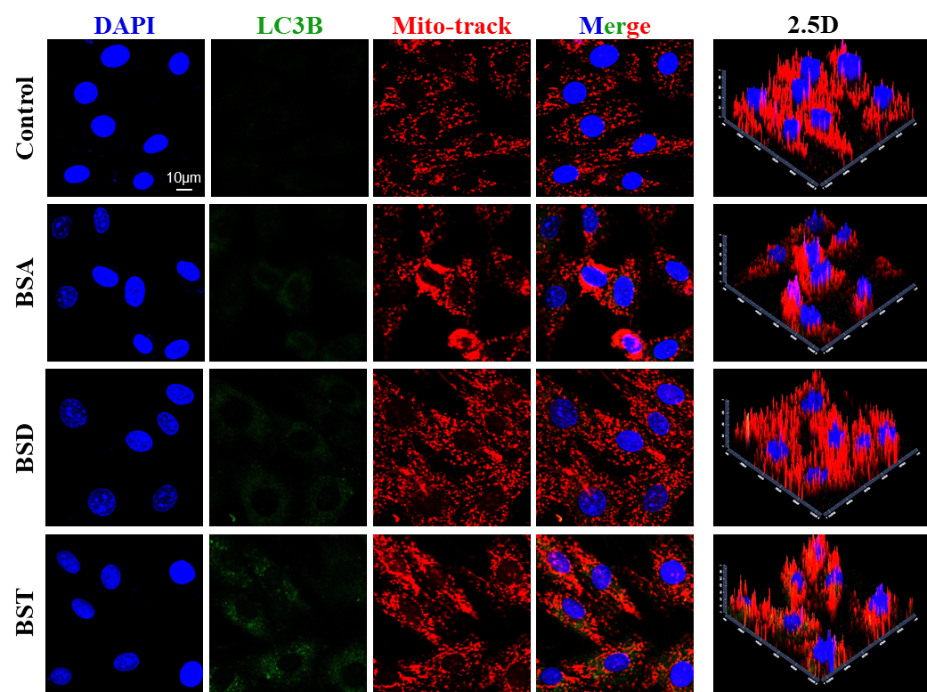


**Figure S18**. Immunofluorescence staining of LC3B in H9C2 cells treated with different enzyme nanogels and incubated under 80 µM H_2_O_2_ and hypoxic conditions for 24 h.


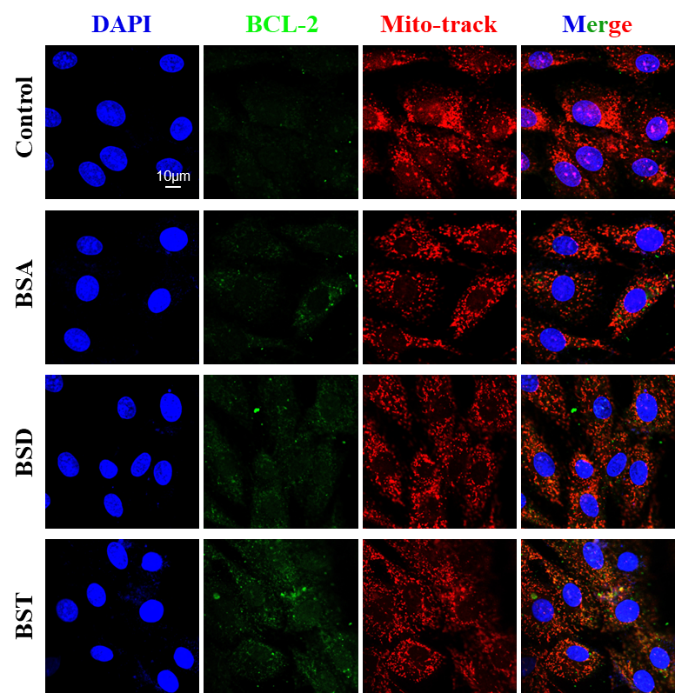


**Figure S19**. Immunofluorescence staining of BCL-2 in H9C2 cells treated with different enzyme nanogels and incubated under 80 µM H_2_O_2_ and hypoxic conditions for 24 h.


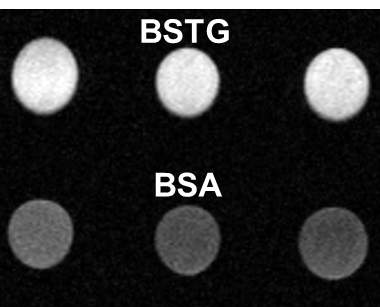


**Figure S20**. MRI images of BSTG hydrogel.


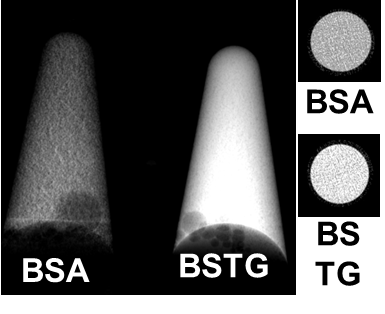


**Figure S21**. CT images of BSTG hydrogel.


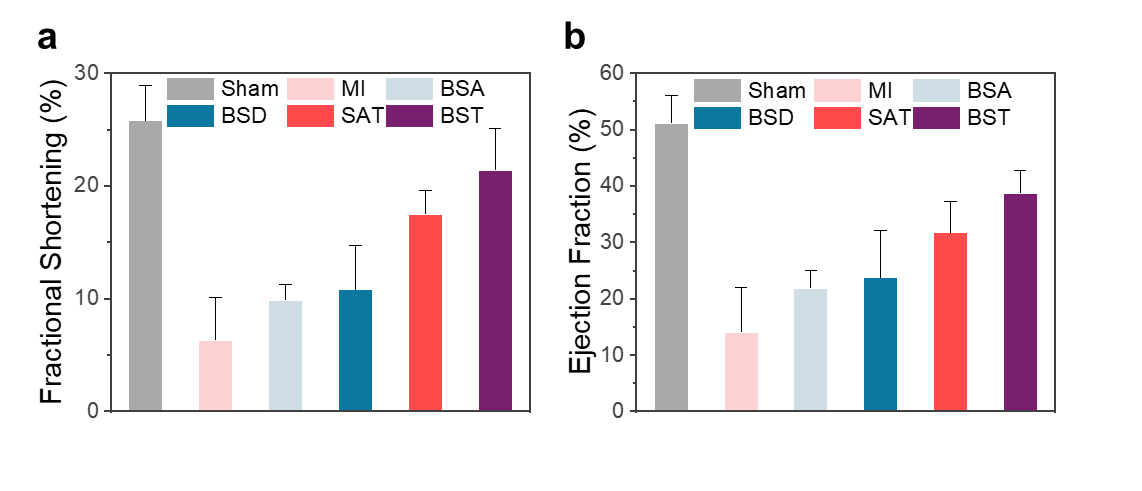


**Figure S22**. (a) Fractional shortening and (b) Ejection fraction assessed by echocardiography 7 days after BST hydrogel treatment (n=5).


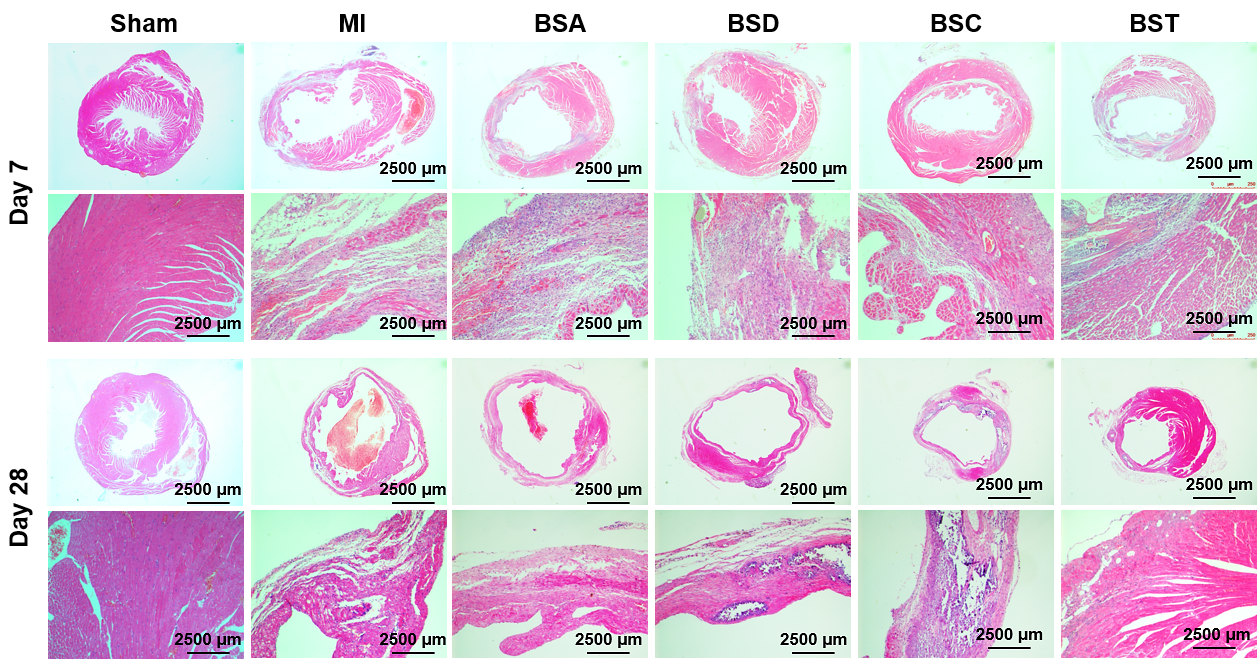


**Figure S23**. H&E staining of myocardial infarction tissue sections of mice after 7 and 28 days of treatment with BSA protein hydrogel loaded with different natural enzyme nanogels.


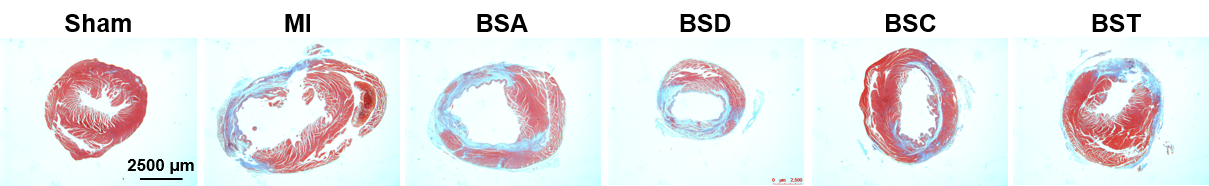


**Figure S24**. Masson staining of myocardial infarction tissue sections of mice after 7 days of treatment with BSA protein hydrogel loaded with different natural enzyme nanogels.


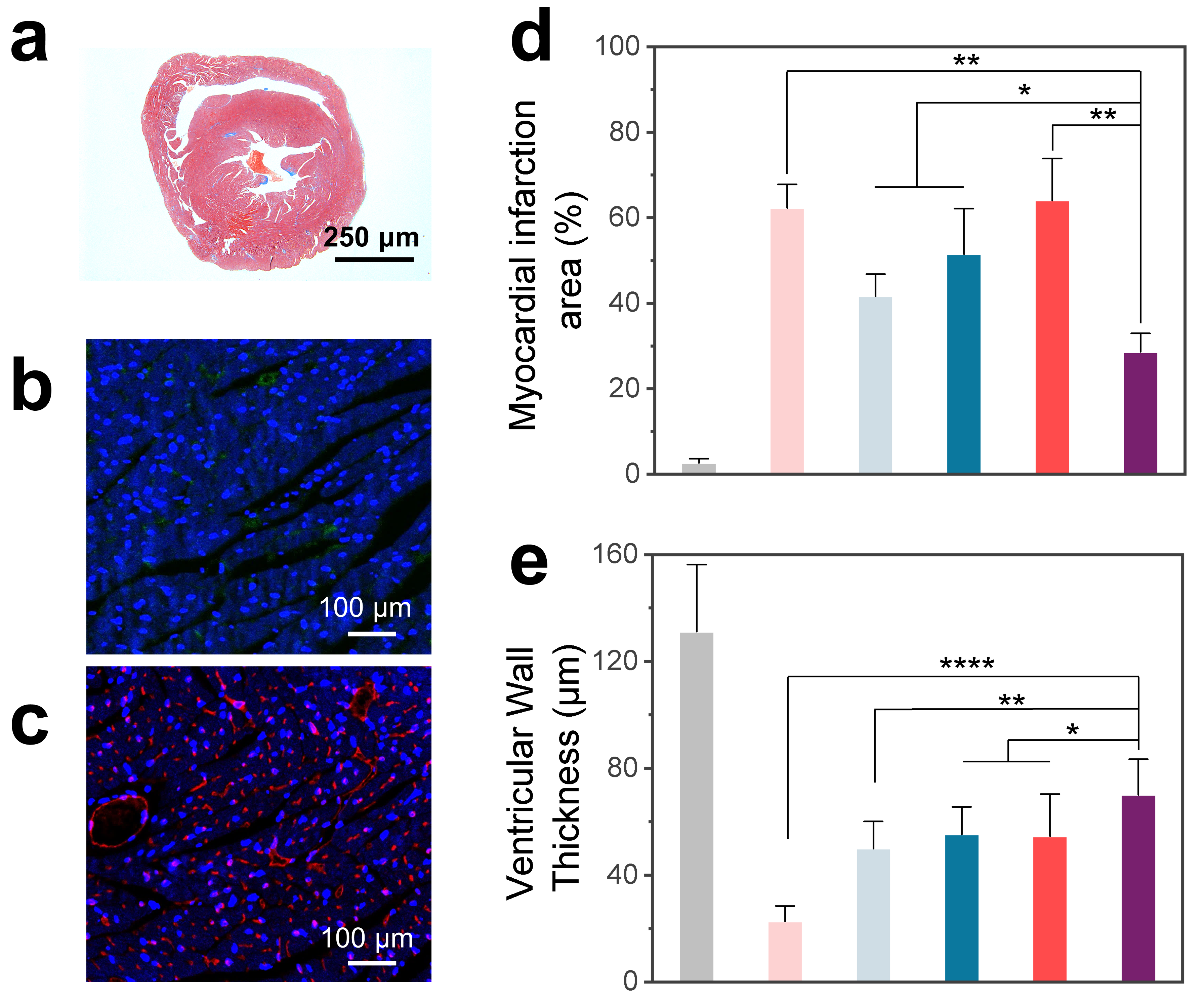


**Figure S25**. After 28 days of Sham treatment, mouse hearts were harvested for (a) Masson staining analysis, (b) HIF-1α and (c) CD31expression. (d) The area of myocardial infarction and (e) the thickness of the ventricular wall in the myocardial tissue after treatment on day 28 (n=5).


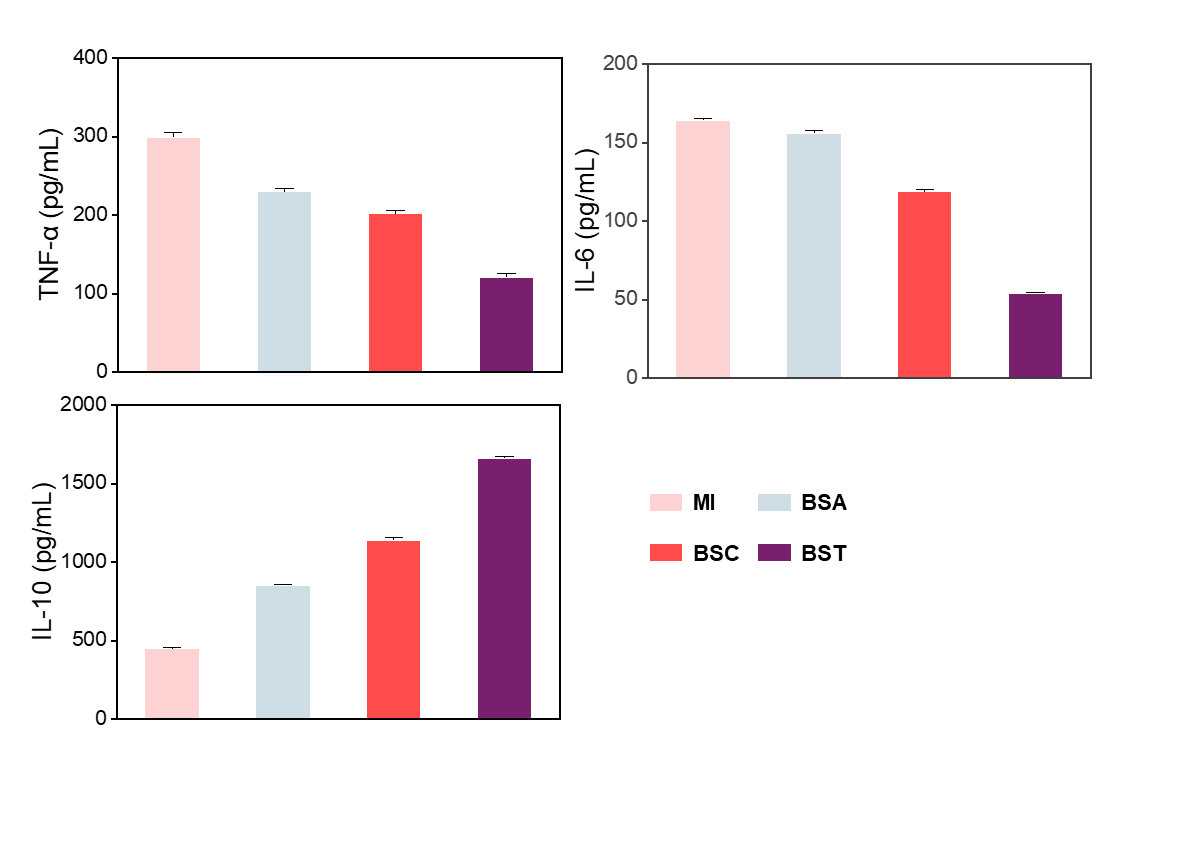


**Figure S26**. After being treated with BSA hydrogels loaded with different natural enzyme nanogels, the release levels of different inflammatory factors: TNF-α, IL-6 and IL-10 (n=3).


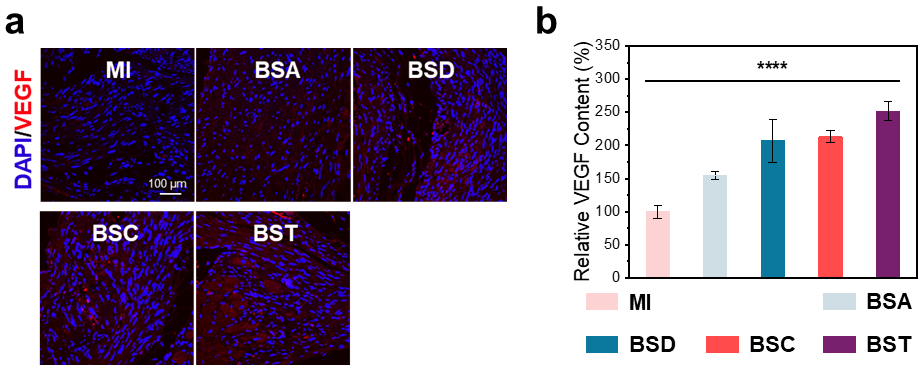


**Figure S27**. After 28 days of BST hydrogel treatment, (a) the mouse hearts were harvested for immunofluorescence staining analysis of VEGF expression levels; (b) Semi-quantitative analysis of the fluorescence intensity of VEGF in Figure S17a (n=3).


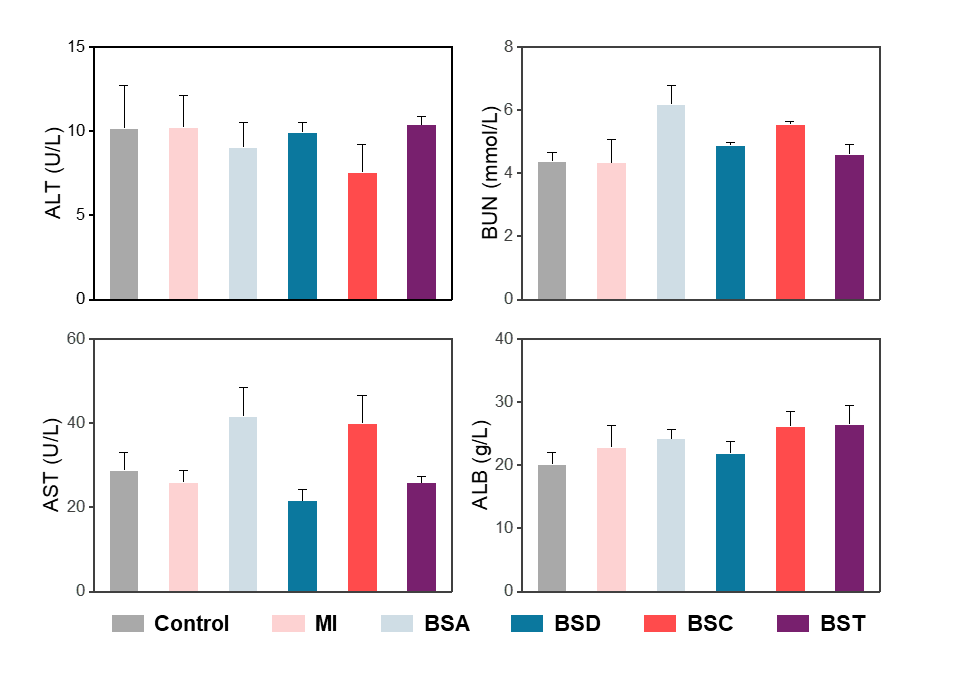


**Figure S28**. Analysis of liver and kidney function in mice with acute myocardial infarction after 7 days of treatment with BSA protein hydrogel loaded with different natural enzyme nanogels (n=5).


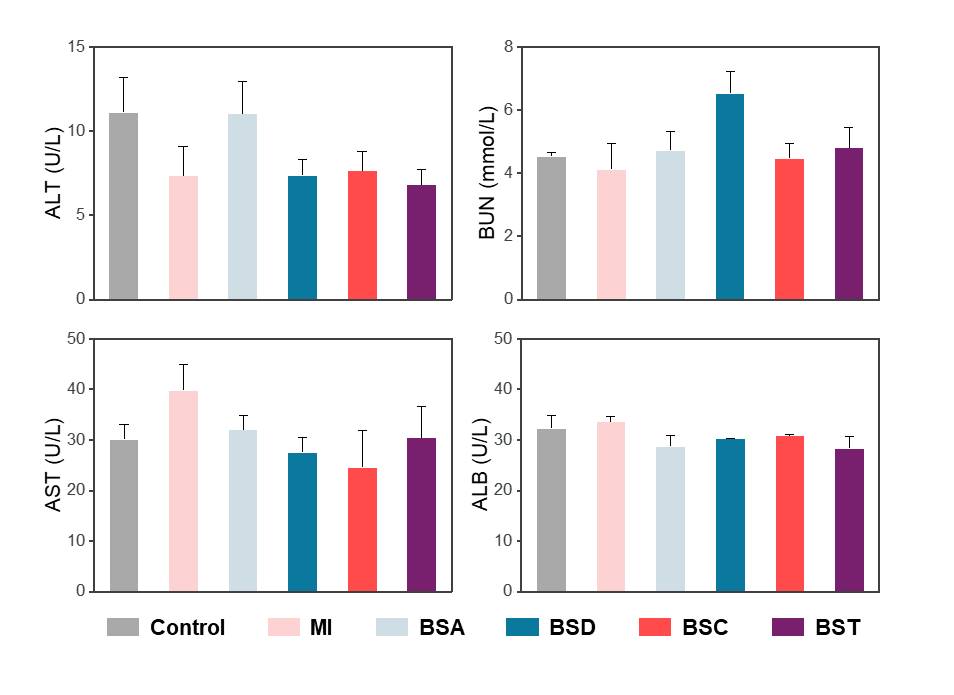


**Figure S29**. Analysis of liver and kidney function in mice with acute myocardial infarction after 28 days of treatment with BSA protein hydrogel loaded with different natural enzyme nanogels (n=5).


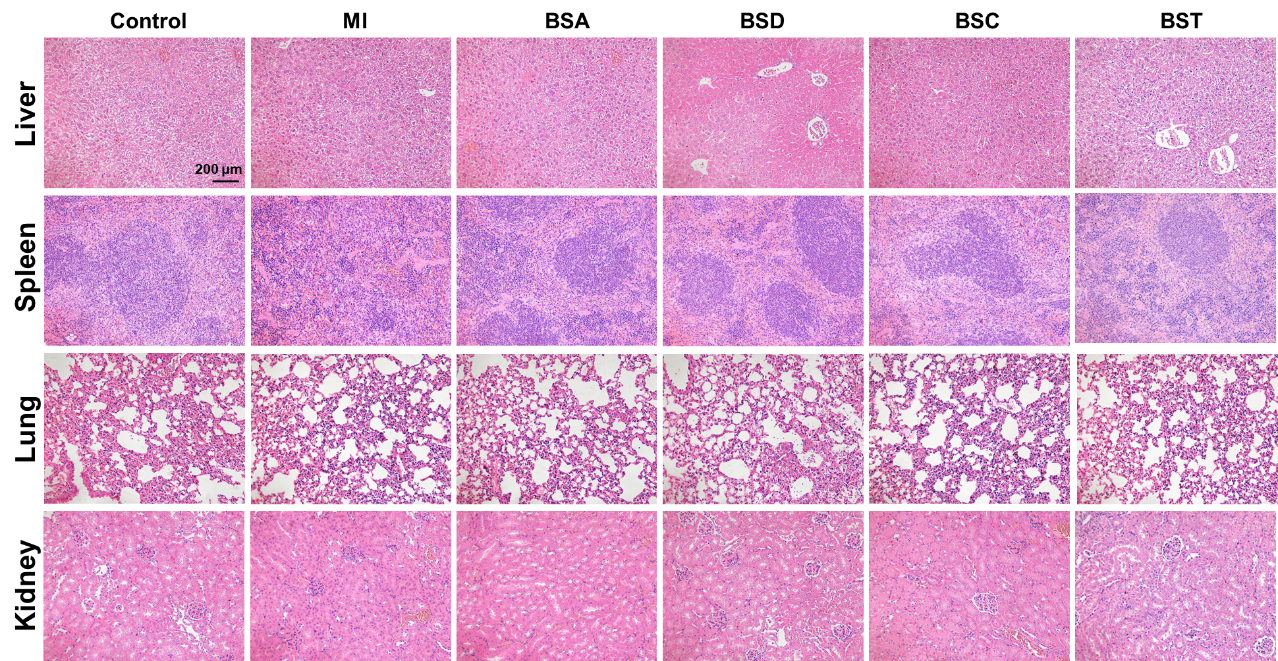


**Figure S30**. Analysis of liver and kidney function in mice with acute myocardial infarction after 28 days of treatment with BSA protein hydrogel loaded with different natural enzyme nanogels.
